# Wandering of the auroral oval 41,000 years ago

**DOI:** 10.1126/sciadv.adq7275

**Published:** 2025-04-16

**Authors:** Agnit Mukhopadhyay, Sanja Panovska, Raven Garvey, Michael W. Liemohn, Natalia Ganjushkina, Austin Brenner, Ilya Usoskin, Mikhail Balikhin, Daniel T. Welling

**Affiliations:** ^1^ Climate and Space Sciences and Engineering Department, University of Michigan, Ann Arbor MI, USA.; ^2^ GFZ German Research Center for Geosciences, Helmholtz Center Potsdam, Potsdam, Germany.; ^3^ Department of Anthropology, University of Michigan, Ann Arbor MI, USA.; ^4^ Finnish Meteorological Institute, Helsinki, Finland.; ^5^ Space Physics and Astronomy Research Unit and Sodankylä Geophysical Observatory, University of Oulu, Oulu, Finland.; ^6^ Department of Automatic Control and Systems Engineering, The University of Sheffield, Sheffield, UK.

## Abstract

In the recent geological past, Earth’s magnetic field reduced to ~10% of the modern values and the magnetic poles shifted away from the geographic poles, causing the Laschamps geomagnetic excursion, about 41 millennia ago. The excursion lasted ~2000 years, with dipole strength reduction and tilting spanning 300 years. During this period, the geomagnetic field’s multipolarity resembled outer planets, causing rapid magnetospheric changes. To our knowledge, this study presents the first space plasma analysis of the excursion, linking the geomagnetic field, magnetospheric system, and upper atmosphere in sequence using feedback channels for distinct temporal epochs. A three-dimensional reconstruction of Earth’s geospace system shows that these shifts affected auroral regions and open magnetic field lines, causing them to expand and wander toward lower latitudes. These changes likely altered the upper atmosphere’s composition and influenced anthropological progress during that era. Looking through a modern lens, such an event would disrupt contemporary technology, including communications and satellite infrastructure.

## Teaser

Earth’s magnetic field weakened severely 41,000 years ago, shrinking the space environment and causing the aurora to wander.

## INTRODUCTION

For over 3.2 billion years, Earth’s intrinsic magnetic field has protected the planet’s atmosphere (*1*) and habitability (*2*) by serving as a shield against the solar wind (*3*), a continuous stream of energetic charged particles emanating from the Sun. This shield, known as the magnetosphere (*4*), takes on a shape resembling a magnetic dipole and is shaped by convective flow processes (*5*) and currents carrying charged particles (*6*). Within the magnetosphere, magnetic field lines transport charged particles by trapping and/or accelerating them, creating a space plasma environment (*7*, *8*) that spans tens to hundreds of Earth radii (*R*_E_; ~6378 km in distance units) in the dayside and nightside, respectively. Earth’s space plasma environment is a complex and nonlinear system that plays a crucial role in safeguarding life from space-based threats (*9*). Charged particles from this environment interact with the upper atmosphere near the magnetic poles, giving rise to the captivating natural light displays known as the aurora borealis (Northern Lights) and aurora australis (Southern Lights) (*10*). Because of their close association with the planet’s intrinsic magnetic field, the attributes of the aurora are directly affected by magnetic disturbances like space storms (*11*), and magnetic substorms (*12*, *13*). These disturbances can alter the trajectories of charged particles, affecting the location and intensity of the aurorae (*14*). Beyond shielding Earth from the solar wind, the space plasma environment also safeguards the planet’s habitability by deflecting harmful solar charged particles and cosmic radiation (*15*), thereby preserving the integrity of the stratospheric ozone layer (*16*) and atmospheric circulation processes (*17*). Furthermore, this magnetic environment plays a critical role in protecting modern technology like satellites (*18*), communication channels (*19*), and electrical power grids (*20*) during such disturbances, underscoring its profound societal importance.

Despite serving as a protective shield, Earth’s intrinsic magnetic field is prone to fluctuations. Owing to its convecting liquid outer core (*21*), which drives the planetary dynamo (*22*), the intrinsic magnetic field has constantly varied in geological time (*23*), occasionally leading to a complete reversal of the field (*24*). On certain occasions, the geomagnetic field changes rapidly over the time span of a few millennia; these events are called geomagnetic excursions (*25*) (henceforth referred to as excursions). Excursions are similar to geomagnetic reversals but occur over shorter timescales (*24*). They cause the intrinsic field strength to diminish and the magnetic tilt to change (*25*), rapidly relocating the magnetic poles over vast distances, even within a human lifetime (*26*). By contrast, the duration of the most recent reversal, Matuyama-Brunhes reversal, is estimated to be in the order of 20 to 30 thousand years (*27*). Although the exact circumstances that cause an excursion are not clearly established (*23*, *24*), geomagnetic records indicate that the Earth’s magnetic field changed markedly about 41,000 years ago (or 41 ka). This event, known as the Laschamps excursion, is the most recent, well-documented, and best-studied global excursion, having been observed in several geological archival records worldwide (*28*). During this event, the axial dipole components of Earth’s geomagnetic field substantially weakened, resulting in a significant reduction in field intensity and a departure from dipolarity (*29*).

The variations observed in Earth’s magnetic field during the Laschamps excursion would have had profound implications on Earth’s biosphere (*30*). The weakening magnetic field intensity likely led to an influx of energetic particles and cosmic radiation penetrating Earth’s atmosphere (*31*), potentially causing notable alterations in atmospheric circulation (*14*) and composition (*32*). Although it is widely believed that these variations had a direct impact on early human development with the emergence of modern humans and megafaunal extinctions being recorded during the same time period as this excursion (*26*), such assumptions were based on oversimplified models of the space plasma environment. Accurately assessing their impact remains challenging without a comprehensive reconstruction of the space plasma environment on a global scale. A previous study (*33*) has attempted to delineate Earth’s magnetospheric morphology and its effect on the upper atmosphere and aurora for nondipolar geomagnetic fields, albeit relying on synthetic data with idealized parameters. Until recently, only a limited number of studies (*34*) have explored the state of the near-Earth space environment concerning transient nondipolar geomagnetic fields. Although these studies provide insights into the effects of geomagnetic reversals on the magnetosphere, the specific conditions of the magnetosphere and aurorae during the Laschamps event have never been investigated until now.

To our knowledge, this manuscript presents the first study that delves into the global repercussions of the fluctuating intrinsic magnetic field on Earth’s magnetospheric structure during the Laschamps event, linking this structure to the formation of a wandering auroral zone. Recent progress in numerical modeling has allowed us to accurately investigate the geospace system not only in three dimensions but also as a collective system. The study breaks down the timeline of the Laschamps excursion into specific temporal epochs that reveal notable variations in the space environment while enabling easy comparisons of variability across different time frames. Moreover, correlating the geophysical findings with anthropological evidences offers a pathway for future research to delve deeper into the precise effects of geomagnetic fluctuations not only on Earth but also on Earth-like planets in distant stellar systems.

## GEOMAGNETIC VARIATIONS DURING THE LASCHAMPS EXCURSION

Recent studies examining the multimillennial variations of Earth’s magnetic field have yielded remarkable insights into the overarching morphology of the Laschamps excursion, suggesting that its genesis lay in the decay and subsequent recovery of the axial dipole field’s influence on the geomagnetic field (*29*, *35*). Studies indicate that the magnitude of the axial dipole field, the field component allowing Earth to have a dipole-like magnetic field structure, directly dictated the scale of the excursion, whether it was regional or global in scope (*36*). Although the field intensity was globally very low, reconstructions of spatial morphology showed that regional field intensities and directions differed strongly (*22*). Notably, the equatorial dipole and nondipole components of the field remained relatively stable amidst these fluctuations (*36*).

**Fig. 1.**
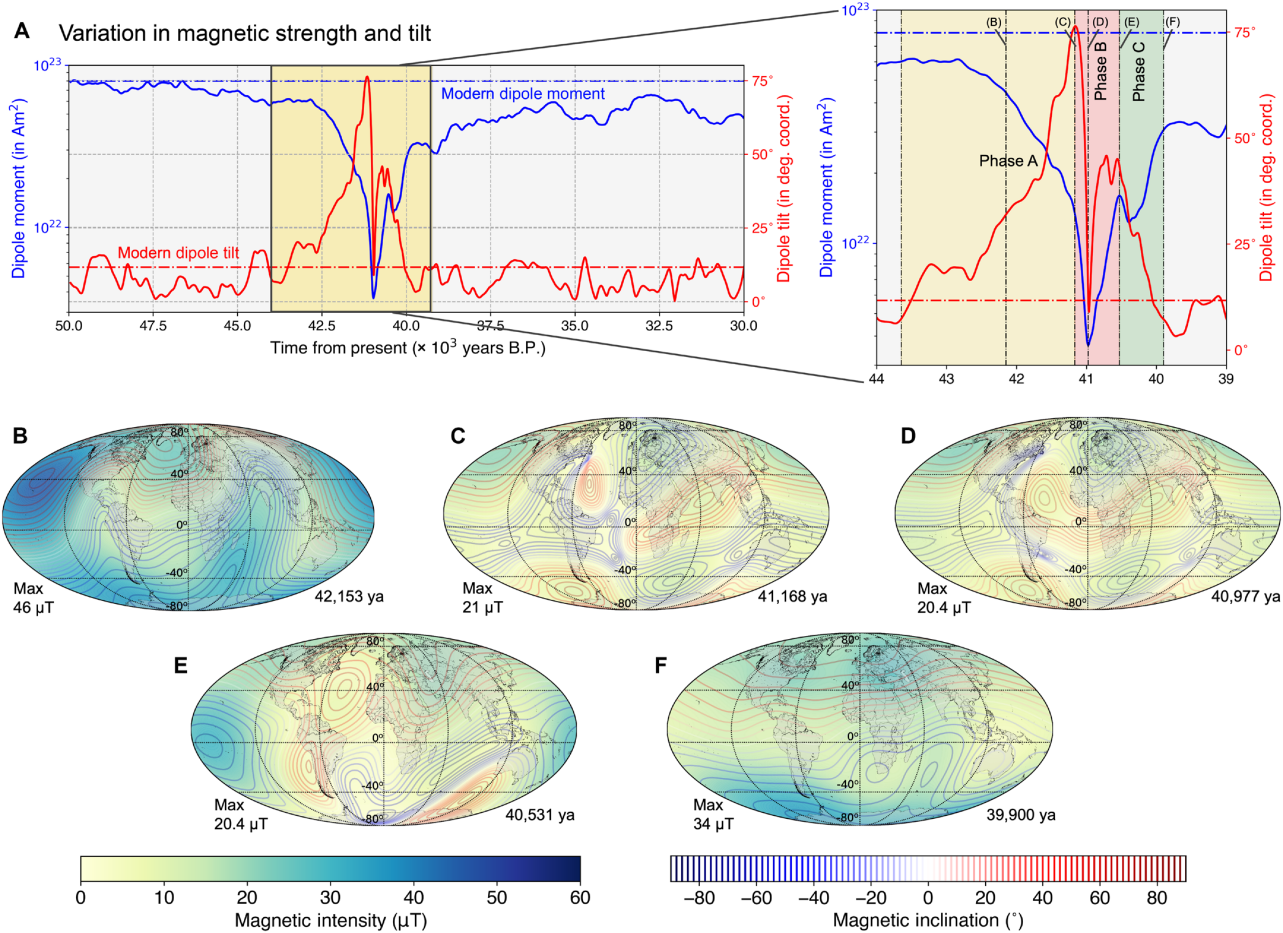
Variations in Earth’s internal magnetic field during the Laschamps Event. (**A**) Intensity (denoted as “magnetic intensity”) and directional variations (denoted as “magnetic inclination”) of the intrinsic magnetic field during the Laschamps excursion, in comparison to modern conditions. B.P., before the present. (**B** to **F**) Global maps of intensity and inclination at the Earth’s surface for selected epochs across the peak field intensity drop during Laschamps as identified in subplot (A).

The Laschamps excursion persisted for roughly 1800 years at the Earth’s surface, and a deeper investigation into the core-mantle boundary across an extended time frame of the event (50 to 30 ka; see Fig. 1A) revealed three distinct periods: pre-Laschamps period (50 to 43 ka), the excursion period (42 to 40 ka), and post-Laschamps period (39 to 30 ka) (*36*). In the pre-Laschamps period, the geomagnetic field resembled the present-day configuration, dominated by a strong axial dipole field with high dipole moment values. However, during the excursion period, the axial dipole field weakened substantially, approaching near-zero levels and occasionally even reversing its polarity for geologically brief periods. Globally, the field intensity plummeted to levels lower than the contemporary field intensity observed over the South Atlantic Anomaly (*37*), the region with the weakest magnetic field strength on present-day Earth. Transitional directional changes in the field were observed worldwide, albeit with varying magnitudes and timings across different regions. Meanwhile, the nondipole field components remained relatively consistent with pre-Laschamps levels. In the post-Laschamps period, whereas the nondipole field continued to behave typically, the axial dipole field began a slow recovery. However, this recovery failed to fully restore the pre-Laschamps levels, resulting in frequent, regionally confined excursions (*36*) until the modern-day field intensity was attained (*24*, *28*). This study focuses its geomagnetic analyses on the period encompassing the peak drop during the Laschamps event, honing in on the excursion state and the brief intervals immediately preceding and following it (see Fig. 1A, inset). Within the 42- to 39-ka time frame, three distinct phases were evident: the stable field before the extreme decay (Phase A), the Laschamps midpoint (Phase B), and the recovery (Phase C).

The differences in the geomagnetic field during the three phases of the Laschamps excursion have been illustrated in Fig. 1 (B to F). Phase A signified a dipole-dominated field with a gradual decline in the dipole moment strength that reached approximately half of present-day values (*38*). Concurrently, as estimated from the dipole components (the first three Gauss coefficients of the geomagnetic field) (*39*), the dipole tilt underwent large deviations from the geographic poles to equatorward latitudes (∼15°; see Fig. 1B). Phase B witnessed the field intensity plummeting to its nadir, with the dipole moment plummeting to approximately an order of magnitude lower than present-day levels (∼10% of the modern dipole moment), alongside rapid and pronounced variations in dipole tilt (see Fig. 1C). These tilt fluctuations stemmed from the reduced axial dipole contribution, resulting in a complex field marked by the emergence of multiple poles, contrasting starkly with a simplistic dipole model (see Fig. 1, D and E). Phase C heralded the beginning of field intensity recovery to moderate levels, with dipole tilt gradually reaching present-day norms. Throughout much of this phase, the field adopted a dipole structure reminiscent of the modern-day configuration (see Fig. 1F). Nevertheless, although the dipole moment at 39.9 ka is similar to that of the pre-Laschamps epoch, discernible differences in the global geomagnetic structure between these two periods were evident, as illustrated by the isoclinic lines on both maps.

## RESPONSE OF THE MAGNETOSPHERIC SYSTEM

Variations in intrinsic magnetic fields have considerable ramifications on a planet’s magnetospheric system. Comparisons between Earth’s magnetosphere and those observed in other planets within the solar system like Jupiter and Neptune show significant disparities in size and structure, primarily attributed to variations in planetary magnetic moments and rotation periods (*40*, *41*). Thus, it is virtually certain that the notable fluctuations observed in the geomagnetic field during the Laschamps excursion would have triggered a marked transformation in Earth’s magnetospheric configuration. Recent investigations into Earth’s magnetospheric structure during the Matuyama-Brunhes reversal—the most recent geomagnetic reversal that took place 778 ka—uncovered a substantial reduction in the magnetosphere’s size and the emergence of numerous regions where the magnetic field lines interact and release energy over a period spanning multiple millenia (*34*). However, because of the accelerated pace of geomagnetic instability characteristic of an excursion, Earth’s magnetospheric configuration transformed profoundly and swiftly over the course of a few centuries during the Laschamps excursion. Leveraging advanced techniques rooted in first principles–based global-scale numerical schemes, we present a three-dimensional (3D) reconstruction of Earth’s prehistoric magnetosphere during the Laschamps excursion and analyze the system’s shape, size, and structure.

**Fig. 2.**
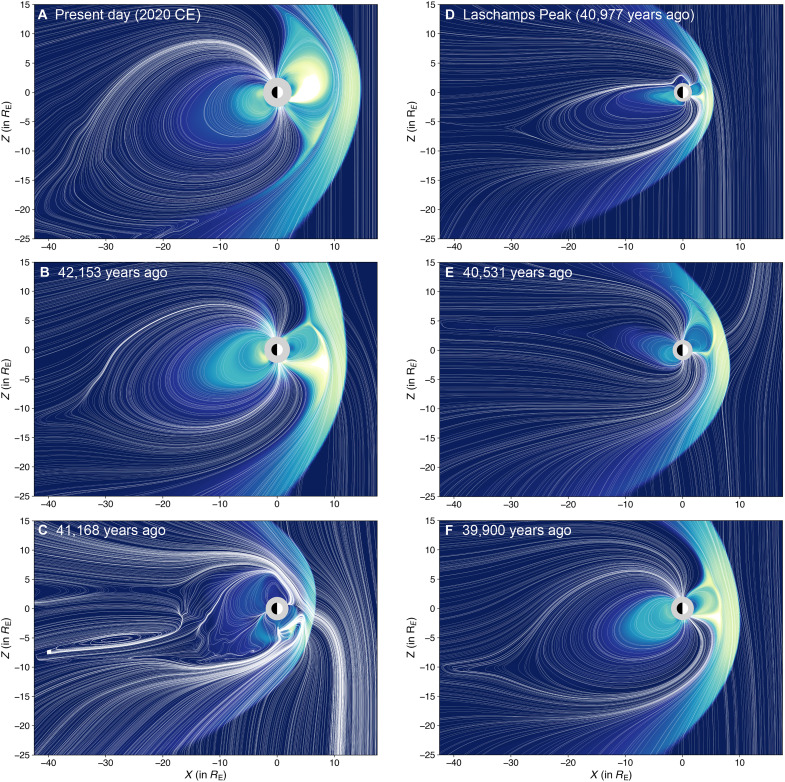
Reconstructed magnetospheric configurations across successive temporal epochs during the Laschamps excursion. (**A**) Present-day magnetosphere at Earth. (**B** to **F**) Magnetospheric morphologies in the *x*-*z* plane (geocentric solar ecliptic coordinates) for temporal epochs spanning the various phases of Laschamps, as delineated in Fig. 1. All configurations were reconstructed under moderately southward solar wind driving conditions at 00:00 UT. White lines trace magnetic field lines, whereas the background contour represents the plasma particle pressure values saturated at 1.5 nPa.

Figure 2 illustrates the swift variations in Earth’s magnetospheric structure across distinct temporal epochs, spanning the various phases of the Laschamps excursion. During much of Phase A of the excursion, Earth’s magnetospheric structure remained largely dipolar, resembling modern times (see comparisons of Fig. 2, A and B). However, a gradual decrease in geomagnetic strength resulted in a reduction in the magnetosphere’s size. By 42.153 ka, Earth’s magnetosphere shrunk to ∼5.3 *R*_E_ (33,804 km from Earth’s surface) on the dayside, almost half the size of the present-day magnetosphere, which ranges between 8 and 11 *R*_E_ (∼51,000 to 70,000 km from Earth’s surface) during moderate solar conditions (*42*). Diminishing geomagnetic strength also expanded the open-closed field line boundary around the poles. The open-closed field line boundary is a region characterized as a boundary between open geomagnetic field lines, magnetic field lines that extend from the magnetosphere into interplanetary space and facilitate the entry of energetic particles from the Sun (*43*) and galactic cosmic radiation (*44*), and closed geomagnetic field lines, looped field lines that connect back to the planetary magnetic field (*45*). Furthermore, a gradual increase in the geomagnetic field’s dipole tilt meant that the magnetosphere’s dipole axis was significantly inclined toward the equator. During this epoch, the magnetosphere tilted by 46.3° to the geographic polar axis, at least four times higher than modern Earth’s geomagnetic tilt of ∼11°. By 41.168 ka (see Fig. 2C), as Phase A of the excursion drew to a close, a weakening axial dipole field caused Earth’s magnetosphere to exhibit strong nondipolar characteristics. The dipole axis was severely tilted to the geographic axis by 76°, resulting in a magnetospheric configuration that resembled those observed in outer planetary systems like Neptune (*46*). Although still displaying dipolar features like a dayside bow shock (*47*) and a compressed magnetosheath region (*48*), the substantial geomagnetic tilt resulted in the open-closed field line boundary relocating near the dayside equatorial magnetospheric boundary. This peculiar magnetic arrangement has been further visualized through 3D snapshots of the prehistoric magnetosphere provided in the Supplementary Materials.

Phase B marked the excursion’s peak alterations to Earth’s magnetospheric structure. By 40.977 ka, the axial dipole strength during this phase was only about 10% of present-day levels. Consequently, the magnetosphere contracted in size, as depicted in Fig. 2D, with the magnetopause—the magnetic boundary of the magnetosphere in the dayside—reaching a meager 2.43 *R*_E_ (15,498 km) from Earth’s surface. On the nightside, the magnetospheric field lines were restricted to ∼32.3 *R*_E_. This phase also gave rise to powerful nondipolar characteristics. Multiple weak magnetic poles emerged around various geographic locations, as illustrated in fig. S4. These poles created clusters of closed field lines that did not extend beyond ∼2 *R*_E_ (12,700 km from Earth’s surface) on both the dayside and the nightside, whereas substantial interactions between open field lines were observed. By 40.531 ka, despite a muted dipole strength (∼19% of modern values), the magnetosphere started to show signs of recovery (see Fig. 2E), with a stronger dayside and nightside closed field line region and a discernible bow shock and magnetosheath region against the upstream solar wind. Notably, the dipole tilt was higher during this epoch, offset by the emergence of nondipolar configurations near the southern geographic pole, leading to a further broadening of the open-closed field line boundary.

As Phase C unfolded and geomagnetic conditions began to recover, Earth’s magnetosphere gradually reverted to its dipolar state (Fig. 2F). By 39.9 ka, the dipole tilt had nearly returned to modern levels (∼10°), albeit with a weaker dipole strength. This resulted in a magnetospheric configuration reminiscent of the pre-Laschamps era yet with a smaller dayside presence and an expanded open field line region near the poles. Notably, closed field line regions expanded on both the dayside and nightside, whereas the bow shock and dayside magnetospheric boundary pushed sunward, extending to 6.4 *R*_E_ (40,820 km). Simultaneously, the nightside magnetosphere enlarged compared to earlier phases (see fig. S5). Toward the latter part of Phase C, there were no notable changes in the dipole tilt angle. Over the subsequent 10,000 years, as the geomagnetic field regained its pre-Laschamps dipole strength, the magnetosphere likely maintained an enlarged open field line region around the poles before gradually shrinking back to the present-day auroral zone.

## GEOLOGICALLY RAPID WANDERING OF THE AURORAL OVAL

The Earth’s magnetosphere is constantly interacting with the solar wind, a stream of charged particles emanating from the surface of the Sun. This dynamic interaction results in the alignment of charged particles with Earth’s magnetic field, which are accelerated in the magnetosphere to precipitate into the upper reaches of the atmosphere (∼110 km). These charged particles, upon collision with neutral atoms within Earth’s atmosphere (*9*), ignite the ethereal display known as the aurorae or the Northern/Southern Lights. Primarily concentrated around the geomagnetic poles, the aurora finds its most pronounced manifestation near the delineating boundary between zones characterized by open and closed field lines (*45*). In doing so, it forms a ring-shaped contour surrounding the geomagnetic poles, commonly referred to as the auroral oval. Variations in magnetospheric shape and structure instigate the auroral oval in both the Northern Hemisphere and Southern Hemisphere to fluctuate. In modern times, the auroral oval’s location, structure, and intensity have been frequently affected by varying solar activity during space weather events (*49*). Space weather studies primarily focus on variations in Earth’s magnetosphere driven by changes in solar wind input to a relatively stable Earth’s magnetic field. In contrast, this study examines variations in Earth’s geomagnetic field under near-constant solar conditions. Building on the magnetospheric variations in the previous section, two substantive changes occurred in the aurora during the Laschamps excursion:

1) With the reduction in geomagnetic dipole moment, the magnetosphere was more compressed. This resulted in the expansion of the polar region encompassed by open field lines and resulted in the subsequent expansion of the aurora (*26*).

2) Rapid variations in the dipole tilt angle over a few centuries enabled the geomagnetic poles to be severely inclined, causing the location of the open-closed field line boundary and, by extension, the auroral oval to wander across the globe.

**Fig. 3.**
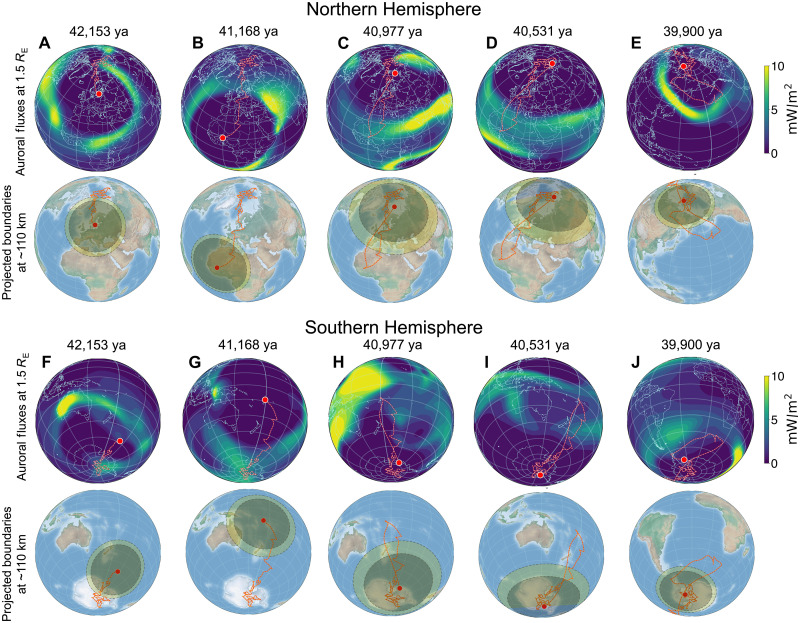
Visualization of auroral charged particle energy flux variations and corresponding auroral zone wandering during the Laschamps excursion. Subplots (**A** to **E**) depict auroral coverage in the Northern Hemisphere at specified temporal epochs as identified in Fig. 1, whereas subplots (**F** to **J**) showcase auroral coverage in the Southern Hemisphere during the same epochs. (Top projection in each subplot) Auroral energy flux contours are represented at 1.5 *R*_E_ (10,000 km), with values saturated at 10 mW/m^2^. (Bottom projection in each subplot) The auroral oval (light green) and aggregate open field line zones (dark green) are projected at atmospheric altitudes (110 km) for each epoch, displayed over an orthographic globe projection. Red lines indicate the trajectory of the geomagnetic poles, based on the axial dipole tilt.

Figure 3 illustrates the transformative shifts across the Northern Hemisphere and Southern Hemisphere auroral zones during the excursion. The contoured rows within the figure delineate the auroral energy fluxes, quantifying the sheer magnitude of energy input from energetic charged particles at a distance of 1.5 *R*_E_ (equivalent to 10,000 km) from Earth’s surface. Concurrently, the approximate positions of the auroral oval and the open-closed field line boundary are mapped at a height of 110 km above Earth’s surface in the subsequent row.

As the geomagnetic dipole underwent a simultaneous weakening and tilting during Phase A, the Northern Hemisphere’s auroral oval traversed from the Arctic region through Western Eurasia to Northern Africa, extending further to Northwestern Sahara. Similarly, in the Southern Hemisphere, the auroral oval shifted from the Antarctic sector toward the eastern expanse of Australia and New Zealand. Notably, the open field line region and the auroral oval underwent a substantial expansion, with the auroral poleward boundary broadening from an average diameter of 5610 km at 42.153 ka to an impressive 8167 km at 41.168 ka. For reference, the modern auroral oval has a diameter of <3000 km during nominal solar wind conditions. During Phase B, this expansion intensified significantly, propelled by the drastic reduction in the axial dipole strength and escalating influence of the nondipolar field. Despite a relatively reduced tilt in the oval, vast expanses of both hemispheres were enveloped by expansive open field line regions, unleashing a substantial barrage of auroral precipitation on a global scale. In modern space weather, extreme events can cause the oval region to expand, but only by a fraction of what occurred during the peak reduction in dipole strength. This epoch witnessed a monumental expansion and the probable fragmentation of the auroral oval, attributable to the nondipolar components of the geomagnetic field. As illustrated in Fig. 3C, the aurora assumed a global presence, engulfing sizeable regions of the Earth with both open and closed field lines, thus sculpting a near-Earth space environment unparalleled in history or during any contemporary space weather phenomenon. This anomalous auroral morphology began its gradual restitution by 40.531 ka, marking the onset of Phase C. The protracted progression of globally unstable auroral zones likely persisted for several centuries until, by 39.9 ka, the Earth’s axial dipole reasserted its dominance, confining the aurora to the polar regions, as is the case today.

## WIDER IMPLICATIONS

The extreme morphological alterations experienced by Earth’s magnetosphere and its auroral zones during the Laschamps excursion likely had substantial repercussions for Earth’s atmosphere and terrestrial environment. Foremost among these was the influx of energetic particles, viz., galactic cosmic rays, solar energetic particles, and solar wind particles, into Earth’s atmosphere. The association between the auroral oval and the open-closed field line boundary is a key factor in quantifying the access of energetic particles to the atmosphere via the geomagnetic cutoff rigidity (*14*, *50*). During the Laschamps event, the cutoff rigidity was substantially reduced due to the weakening of the geomagnetic field (*51*), which also affected the intensity and spread of the auroral zone. For instance, during Phase B’s nondipolar configuration of the magnetic field, precipitation of energetic particles into the upper atmosphere likely increased on a global scale, generating aurorae over a significantly large area despite the possible absence of strong magnetospheric drivers like the ring current or trapped-particle radiation belts (*34*). This illustrates the influence of global geomagnetic conditions on local auroral phenomena and ground-level cosmic ray–induced radiation during the Laschamps event. Studies of the effects of cosmic radiation during paleomagnetospheric polarity transitions (*31*) reveal that, with a reduction in dipole moment strength, lower latitudes including the tropical zone become accessible to high fluxes of lower-energy particles, ushering in zones of impact for diverse magnetospheric configurations. Cosmic radiation is the source of cosmogenic nuclide production in the atmosphere and for ozone generation in the stratosphere and mesosphere (*52*, *53*). A surge in energetic particle influx brought about by the magnetospheric and auroral conditions during the Laschamps excursion would have engendered altitudinal variations in atmospheric circulation (*15*), potentially causing marked shifts in global atmospheric ionization and circulation (*26*). Earlier studies were based on an unrealistic assumption of the total absence of the geomagnetic field. Here, we provide more realistic results for the geomagnetic shielding pattern during the Laschamps excursion making a basis for further more detailed modeling of the atmospheric effects.

Conceivably, the reduction in cutoff rigidities and ensuing surge in cosmic radiation–driven ozone depletion during the Laschamps event affected life on Earth, including humans. Recent studies show substantial oxygenation of the atmosphere and, accordingly, radiation-induced changes in macroscopic fauna’s physiologies (*54*). To refine existing estimates of radiative dosage at Earth’s surface during the Laschamps excursion (*33*, *53*), further investigation should incorporate the geophysical dynamics demonstrated in this study. Nonetheless, evidence from cosmogenic radionuclides indicates a notable peak in cosmic radiation in the Earth’s atmosphere during the Laschamps event. Of particular interest here are higher-than-present doses of harmful ultraviolet radiation (UVR) and their potential effects on humans, especially in areas of open field line coverage (*26*, *55*).

**Fig. 4.**
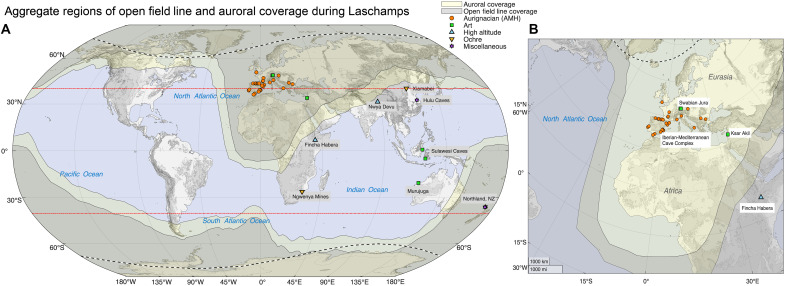
Map of the combined coverage of the auroral oval (yellow-shaded area) and open field line regions (gray-filled) throughout the Laschamps event (42.153 to 39.9 ka). Anthropological activity during this period is highlighted on (**A**) a global scale and (**B**) on the Northern Hemisphere (Europe-Maghreb sector). The legend is as follows: Orange circles denote sites associated with evidence of the Aurignacian toolkit. Green squares denote locations with evidence of cave art and portable art. Yellow inverted triangles indicate the presence of prehistoric ochre mines. Light blue triangles signify high-altitude sites. Purple stars denote miscellaneous evidence of heightened radionuclide production. The dotted black line indicates estimates of the present-day auroral boundaries under quiet solar conditions. The red line represents previous estimates of the auroral boundaries during the Laschamps event (*26*).

The Laschamps excursion coincides with several human behavioral and technological changes that could reflect efforts to minimize exposure to UVR. Figure 4 illustrates the combined coverage of the auroral zone and open field lines throughout the Laschamps event and indicates concurrent anthropological activities that may reflect human responses to near-surface changes during the excursion. Preliminarily, we focus on Western Eurasia, which not only experienced prolonged open field line and auroral coverage (~3 ka) but also has an extensive record of human activity before, during, and following the Laschamps event. Other world regions—such as the poles, Americas, West Africa, and the Maghreb—were also within the region of open field line coverage for much of the event, but evidence for human occupation during the excursion is sparse or equivocal (*56*). Neanderthals (*Homo neanderthalensis*) emerged at least 300 ka and inhabited Eurasia until roughly 40 ka (*57*); their disappearance is coincident with the terminal Laschamps. Evidence suggests anatomically modern *Homo sapiens* (AMH) were in Europe as early as 56.8 ka (*58*) and dispersed rapidly across the region between Bulgaria and Portugal roughly 45 ka (*59*).

In humans, overexposure to UVR is linked to sunburn, carcinogenesis, immunosuppression, ocular pathology/blindness, and folate depletion, which is associated with congenital disorders and infant mortality (*60*–*62*). Genetic evidence suggests that both Neanderthals and AMH living in Europe during the peri-Laschamps exhibited richly pigmented skin, hair, and eyes, which correlates with photoprotection (*63*). All shades of skin pigmentation are nonetheless susceptible to detrimental effects of UVR (*64*). Increased UVR during the Laschamps event, particularly in areas of open flux coverage, could have increased rates of sunburn, vision impairment, infant mortality, and lethal melanomas (*65*).

Roughly 43 ka, the Aurignacian cultural complex—a suite of artifact forms and types generally associated with AMH—was evident across much of Western Eurasia (*59*). Ochre (hematitic iron oxide) is a common component of the Aurignacian toolkit and has demonstrated efficacy as a topical photoprotector (*66*). The mineral’s increased frequency in archaeological sites dating to the peri-Laschamps could be due in part to its use as a sunscreen (*67*). Moreover, the Aurignacian cultural complex includes tools associated with the production of tailored clothing (i.e., garments fitted to the limbs), including stone scrapers and blades (hide preparation) and awls and needles of bone, antler, or ivory [garment construction; (*68*)]. Although both Neanderthals and contemporary AMH produced technologies associated with clothing manufacture, only AMH appear to have produced technologies consistent with the manufacture of tailored clothing (*69*); Neanderthals are assumed to have produced only relatively simple, draped clothing (e.g., capes). The tailored clothing produced by AMH would have allowed greater freedom of movement than draped clothing, maintaining body coverage while preserving range of limb motion, and permitting people to stray farther and longer from shelters (*70*). Neanderthals’ decline was almost certainly multifactorial, but it is possible that topical sunscreens and tailored clothing provided AMH essential photoprotection and access to resources in places and at times they would otherwise have been inaccessible (*71*), a competitive advantage in an environment characterized by volatile climate including very cold conditions and probably also heightened risks from UVR exposure during the Laschamps.

Others (*26*) have noted co-occurrence of the Laschamps with the earliest known representational cave art—which depicts animals, anthropomorphs, and other figures or scenes, as opposed to abstract marks or designs—including images of animals in eastern Borneo, Indonesia (*72*) and Western Australia, and a hunting scene in southwestern Sulawesi, Indonesia (*73*). To this, we add that the Laschamps event coincides with early examples of portable art (*74*) and musical instruments (*75*). In addition, two of the earliest known high-altitude sites—Fincha Habera in the Bale Mountains of Ethiopia [~3500 m above sea level; (*76*)] and Nwya Devu on the Tibetan Plateau [~4600 m above sea level; (*77*)]—were in use during the Laschamps. Of course, it is possible that these behaviors were simply part of a new cultural repertoire, unrelated to the geomagnetic excursion (i.e., to changes in game availability or visibility of aurorae), but spatiotemporal coincidence of these cultural phenomena with Laschamps-induced changes in auroral visibility and open field line coverage are compelling and warrant further investigation to validate and clarify the correlation.

Considering the probable impact of the Laschamps excursion on early humans and their way of life, a similar event today would likely have dire consequences for modern humans. Despite the gradual nature of the geomagnetic variations, they were more extreme than those caused by the strongest space weather events on record (*78*). The ramifications of a Laschamps-like magnetospheric configuration and auroral oval would reverberate across all facets of modern communication, satellite infrastructure, and intercontinental travel. Although objects in low Earth orbit, such as the International Space Station, would remain shielded from solar events by the weakened magnetosphere, communication satellites (typically orbiting at a height of 6.6 *R*_E_ or 42,000 km from Earth’s surface) would endure severe disruption, necessitating enhanced shielding to safeguard internal electronics from solar energetic particles and galactic radiation. Moreover, the current reconstruction of the magnetosphere does not account for the impact of extreme space weather events, which could potentially render Earth’s magnetosphere and auroral oval susceptible to tumultuous interactions with the solar wind even during nominal space weather occurrences, resulting in widespread technological failures of both spaceborne (*16*) and terrestrial infrastructure (*18*). Navigation techniques and communication systems would frequently falter during such episodes (*17*), exacerbating climatic perturbations (*79*). Although the threat of an excursion is not imminent, the geomagnetic dipole field has been tilting in recent years (*80*) and has steadily declined by 1% every two decades for the past 180 years (*29*). This underscores the critical importance of understanding consequential variations in the magnetospheric system and associated geomagnetic phenomena like the aurora, which serve as vital bulwarks in preserving the long-term viability of hosting life in planetary environments (*81*).

## DISCUSSION

The Laschamps excursion marked a distinct episode in Earth’s magnetic history. Over the course of a millennium, the axial dipole experienced a precipitous decline, resulting in a drastic reduction in geomagnetic field strength to a mere 10% of present-day levels and the poles tilting by over 75° relative to the geographic axis. During the height of the excursion, Earth’s magnetic field displayed a highly nondipolar configuration, gradually recovering over at least the next 10 millennia to its present-day state. To our knowledge, this study presented the first reconstruction and subsequent analysis of the global space environment during this time frame and drew the following conclusions:

1) The Laschamps event profoundly affected Earth’s magnetosphere. The decline of the axial dipole field led to a contracted space plasma environment which extended to only 15,500 km from Earth’s surface on the dayside at the height of the excursion. As the field assumed a more nondipolar configuration, the magnetosphere exhibited multiple magnetic poles, experienced a substantial expansion of the open field line regions, and underwent a marked tilt in the geomagnetic axis, which altered the morphology of open and closed field lines. Although recovery of the magnetosphere back to a dipolar morphology was relatively swift, lasting only a few centuries, the restoration of the present-day structure and size would require at least another 10,000 years.

2) The variations in the magnetosphere altered the formation of the auroral zones, which expanded due to the contracted size of the magnetosphere and the enlarged open-closed field line region. As the excursion unfolded, the pronounced tilt in the geomagnetic poles caused the aurorae to wander toward lower latitudes in both hemispheres. Furthermore, the emergence of a nondipolar magnetic field led to the proliferation of an expanded, more globally distributed auroral zone that affected the middle and lower latitudes more prominently. The gradual recovery in the relocation of auroral zones is discernible by 39.9 ka as the axial dipole gradually regained its strength.

3) The proliferation of open field lines, driven by shifts in magnetospheric morphology and the migration of the aurora, undoubtedly resulted in heightened penetration of energetic radiation from outer space. Notably, the areas most affected by open field lines align with significant anthropological change, including behavioral and technological adjustments that may reflect efforts to minimize exposure to UVR.

In summary, this study offers a previously unobserved glimpse into Earth’s space environment shaped by a weakened magnetic field with prominent nondipolar components. Although the implications of space weather highlighted in this research are pivotal for comprehending and forecasting potential events that could affect humanity, the investigation also presents a fascinating portrayal of the intricate interplay among Earth’s geophysical systems, which are essential for sustaining life on the planet.

## MATERIALS AND METHODS

The following sections describe the numerical methods used in this study to reconstruct the paleo-space environment. In brief, three models are used to reconstruct and analyze the intrinsic magnetic field, the magnetosphere, and the aurora. The underlying principles of the numerical models and their usage in this investigation are described in the following.

### Paleomagnetic field models

Reconstructions on the Earth’s magnetic field variations on long multimillennial timescales are important to understand the source of the geomagnetic field and its effects on the environment and climate. Data that provide information on the paleomagnetic field come from geological archives, volcanic rocks, and sediments. Continuous progress in compiling new data enables us to model the field on even longer timescales, from human civilization to millions of years (*82*). The paleomagnetic field is often studied with paleo-intensity stacks or models of dipole moment variations (*83*), which provides information on the global intensity only. Several global models, produced over the past decade, cover the Laschamps excursions and provide a robust picture of the global field morphology (*21*). Recent models (*29*, *35*) made use of significantly increased dataset, with more strict criteria of selecting data. All these models suggest that the Laschamps excursion is mainly driven by axial dipole decay and recovery, without any notable changes in the nonaxial dipole terms (equatorial dipole and nondipole components). For the purpose of extracting the robust characteristics of the Laschamps excursion, a few models were developed to test the effects on data selection and age models (*29*). This study uses the LSMOD.2 (*29*) model, built from regionally stacked records and regionally aligned by intensity variations. The reconstructed variations agree well with the other models (*35*) based on different datasets, which makes the model reliable and useful for studying the paleomagnetosphere and past auroras.

### BATS-R-US global MHD model

The magnetospheric reconstruction was conducted using the Block-Adaptive Tree Solar wind Roe-type Upwind Scheme (BATS-R-US) global magnetohydrodynamic (MHD) model (*84*). The BATS-R-US model solves the ideal single-fluid MHD equations using a finite-volume approach to create the near-Earth space plasma system in a 3D numerical environment. The computational domain for Earth-based scenarios of BATS-R-US extends from 32 *R*_E_ upstream to the solar wind (dayside) to ∼224 *R*_E_ downstream (nightside) in the *x* direction and ±128 *R*_E_ in the *y* and *z* coordinates (GSM). The inner boundary of the simulation domain is set at 1.5 *R*_E_ (10,000 km) from the Earth’s surface. BATS-R-US uses a flexible, block-adaptive Cartesian grid that reserves the highest resolution to regions of interest, ensuring the best combination of performance and accuracy. The adaptive grid used in this study has an initial spatial resolution of 1/16th *R*_E_ (400 km) around the inner boundary of the simulation domain, with a grid resolution increasing to a maximum of 8 *R*_E_ (50,000 km) as one moves farther away into Earth’s nightside (>100 *R*_E_). BATS-R-US is typically used as part of a Space Weather Modeling Framework (*85*), which allows multiple models to be coupled to each other. This model version uses a multipolar terrestrial geomagnetic field as input and allows ionospheric and/or upper atmospheric models to use MHD variables and compute upper atmospheric quantities (*33*, *34*).

### MAGNIT auroral precipitation model

The MAGnetosphere-Ionosphere-Thermosphere (MAGNIT) auroral model (*86*) computes the strength and global structure of the aurora by evaluating the flux contributions of individual sources of aurora. MAGNIT uses adiabatic kinetic theory to compute auroral energy fluxes from MHD state variables like plasma pressure, density, and temperature. MAGNIT has been designed to simulate the impact of space weather events on auroral formation and has previously been validated against existing auroral models for real-life space weather events (*87*). In this study, MAGNIT has been used to estimate contributions from three sources of auroral precipitation: electron diffuse, ion diffuse, and monoenergetic. The MHD model computes particle density and temperature, which are then used to parameterize electron and ion energy fluxes. An electron-to-ion temperature ratio of 1:5 is assumed (*88*). Monoenergetic energy fluxes are computed as a function of current parallel to the magnetic field using the Knight-Friedman-Lemaire method (*89*, *90*) by assuming a potential drop along a magnetic field line. Although MAGNIT is capable of calculating additional auroral sources (e.g., broadband ionization and polar cap rain), these sources have limited impact on the total auroral energy flux and therefore have not been included. MAGNIT’s auroral energy fluxes were determined at the same altitude as the inner boundary of the global MHD simulations and then empirically mapped down to the altitude of the upper atmosphere.

### Modeling setup and caveats

Conducting a numerical study of a complete excursion is numerically expensive. Therefore, unique temporal epochs were chosen to model the global geomagnetic conditions across the spectrum of changes that occur over the ∼1300 years spanning the multiple phases of the excursion. Specifically, five temporal epochs—42.153, 41.168, 40.977, 40.531, and 39.900 ka—were chosen across the span of Phases A, B, and C of the excursion. These epochs signify unique conditions in both the intrinsic and global geomagnetic conditions and helped identify the key variations in the magnetosphere and the auroral structures. The simulations were carried out using a coupled multimodel approach. The three aforementioned models—LSMOD.2, BATS-R-US, and MAGNIT—were combined to form a one-way feedback chain, such that outputs from one model were used as inputs for the next.

The LSMOD.2 model was used to simulate the global paleomagnetic reference field at the chosen epochs. The reference field was formatted as a set of spherical harmonic variables, similar to the International Geomagnetic Reference Field (IGRF) Model (*38*), the standard magnetic model used for modern Earth.

The geomagnetic reference field was then used as the inner boundary condition for BATS-R-US. Simulations of BATS-R-US were carried out for each of the five temporal epochs. BATS-R-US requires input information in the form of solar wind plasma description, which serves as the driving condition for the simulations. Because solar plasma variations were limited in comparison to modern day (*91*), modern defaults for a quiet-time solar wind condition with a bulk speed of 400 km/s and a southward magnetic field component of 5 nT in the *z* direction were used. These conditions were run from 00:00 to 03:00 UT (250,000 iterations). The inner boundary of BATS-R-US assumes an ionospheric shell with a number density of 28 cm^−3^ but is not coupled to a dedicated ionospheric solver. Furthermore, these simulations do not consider the impacts of extreme space weather conditions and diurnal variations on the global magnetosphere. These aspects of the magnetospheric domain would be investigated in a future study.

MHD output variables mapped to the inner boundary of BATS-R-US were used by MAGNIT to compute multiple sources of auroral precipitation. BATS-R-US outputs like plasma pressure, density, and magnetic field were used to compute parallel currents and plasma temperatures at the inner boundary of the MHD model (1.5 *R*_E_). These values helped discern the general structure of the aurora. The latitudinal-longitudinal extent of the aurora was mapped down to atmospheric altitude (∼110 km) by assuming a dipole configuration for the geomagnetic field and tracing field lines between these altitudes. To do this, the energy flux and average energies of the auroral charged particles were computed over a spherical grid situated at the inner boundary of BATS-R-US. Using geomagnetic coordinates, the values were mapped down to atmospheric altitudes following dipolar magnetic field lines. A dipolar magnetic field line can be described as follows (*7*)

$$r=LR_{E}\left(\Lambda \right)$$ (1)

where *L* is the L-shell and Λ is the magnetic latitude of the given field line. Because the field line (L-shell) would remain constant, the mapping along a field line is done in the following manner

$$\frac{r_{\text{IB}}}{\left(\Lambda_{\text{IB}}\right)}=\frac{r_{110}}{\left(\Lambda_{110}\right)}$$ (2)

where *r*_IB_ is the radial distance at the inner boundary of the MHD model, *r*_110_ is the radial distance at the atmospheric boundary, Λ_IB_ is the magnetic colatitude at the inner boundary of the MHD model, and Λ_110_ is the magnetic colatitude at the atmospheric boundary. Work is currently in progress to enhance the precision of mapping field lines from the inner boundary of BATS-R-US to the upper atmospheric altitude in MAGNIT. This entails integrating a multipolar setup capable of accommodating nondipolar magnetic configurations, which will enable more realistic computation of auroral fluxes along nondipolar magnetic field lines. This approach would eliminate the need to estimate auroral flux values at upper atmospheric altitudes based on a dipolar configuration, thereby minimizing uncertainties and errors. This is beyond the scope of the current investigation and will be presented in a future study.

## Supplementary Materials

### This PDF file includes:

Supplementary Text Figs. S1 to S14 References
