# Supplementary material for: Wandering of the auroral oval 41,000 years ago
Source: Sci Adv. 2025 Apr 16;11(16):eadq7275. doi: 10.1126/sciadv.adq7275 (PMC12002135; doi:10.1126/sciadv.adq7275)
Supplement: Supplementary file 1 — Supplementary Text Figs. S1 to S14 References [file sciadv.adq7275_sm.pdf]

Science Advances
AAAS

Supplementary Materials for

Wandering of the auroral oval 41,000 years ago

Agnit Mukhopadhyay *et al.*

Corresponding author: Agnit Mukhopadhyay, agnitm@umich.edu

Sci. Adv. **11**, eadq7275 (2025)
DOI: 10.1126/sciadv.adq7275

This PDF file includes:

Supplementary Text
Figs. S1 to S14
References

Supplementary Text

Anthropological Analysis

Hypothesized near-future excursions or reversals (*67, 83, 92 - 94*) have generated interest in biospheric and other effects of global-scale geomagnetic events (*95*). Past geomagnetic reversals have been linked to mass extinctions, speciation events, and other evolutionary phenomena (*83*), and multidisciplinary studies are crucial for understanding relationships between the geomagnetic field and human biocultural and technological systems.

While more simulation and experimental studies are also necessary to better understand the interplay between the geomagnetic field and near-Earth atmosphere, it seems likely that the geomagnetic field is implicated in Earth's climate systems (*96, 97*). During the Laschamps, open field lines may have resulted in higher-than-present doses of harmful ultraviolet irradiation (UVR), particularly in areas of open flux coverage (*26, 55*). Our model indicates that open flux coverage and, therefore, potentially intense UVR, was of longer duration in western Eurasia than in other world regions during the Laschamps event.

Neanderthals (*Homo neanderthalensis*) emerged at least 200 ka and inhabited Eurasia until roughly 40 ka (*57*); their disappearance is coincident with the terminal Laschamps. Evidence suggests anatomically modern Homo sapiens (AMH) were in Europe as early as 56.8 ka (*58*) and dispersed rapidly across the region between Bulgaria and Portugal roughly 45 ka (*59*). If UVR was higher than present during the Laschamps, both Neanderthals and AMH would have experienced detrimental effects including inflated rates of infant mortality, visual impairment, and lethal melanomas (*65, 98, 99*). Shelter, including caves, can provide partial protection from harmful UVR, which could account for the reported increase in cave use during the Laschamps (*26*). However, exposed tissues remain susceptible even under cover because UVR is easily scattered and reflected (*100*). Clothing can be an excellent barrier, augmenting UVR protection while in shade and providing protection when shade is unavailable or incompatible with other activities such as procurement of essential resources (water, food, and fuel for fires).

Roughly 43 ka, a common "technocomplex"—a suite of artifact forms and types collectively referred to as Aurignacian and generally associated with AMH—was evident across much of western Eurasia (*59*). The Aurignacian technocomplex is characterized in part by tools associated with the production of tailored clothing (i.e., fitted to the limbs), including stone scrapers and blades (hide preparation) and awls and needles of bone, antler, or ivory (garment construction; *68*). Stone scrapers are also ubiquitous at contemporaneous archaeological sites attributed to Neanderthals, and Neanderthals are particularly associated with the so-called Lavallois technique of stone flake production, which maximizes useful scraping edges. Still, Neanderthal sites lack other sorts of tools associated with clothing production, and they are assumed to have produced only relatively simple, draped clothing (e.g., capes; *69*). The tailored clothing produced by AMH, conversely, would have allowed greater freedom of movement than draped clothing, maintaining body coverage while preserving range of limb motion, and permitting people to stray farther and longer from shelters (*70*). In these ways, tailored clothing provides access to resources in places and at times they would otherwise be inaccessible (*71*), a competitive advantage in an environment characterized by volatile climate including very cold conditions and probably also by heightened risks from UVR exposure during the Laschamps.

Ochre (hematitic iron oxide) is also a common component of the Aurignacian toolkit. Ochre has a wide range of known uses, including as a paint applied to bodies and other surfaces (e.g., cave walls), in adhesives, as a substance for tanning hides, and as an insect repellent. It is also traditionally applied as a form of sunscreen in some parts of Africa, and its efficacy in this capacity has been confirmed by in vivo tests of the compound's sun protection factor (SPF; *66*). Investigations note that the increased frequency of ochre in archaeological sites dating to the peri-Laschamps may indicate its use as a sunscreen (*67*). Also noteworthy in this regard are an ochre mine in Eswatini (Swaziland), where large-scale extraction of iron oxides began 42 ka (*101*), and an ochre processing station in northern China dating to ~40 ka (*102*). The processing station is "most likely" associated with AMH, but may have been produced by Neanderthals or Denisovans, and the balance of the toolkit does not resemble Aurignacian ones. It is therefore possible that this site's occupants did not produce fitted clothing and used ochre sunscreen instead. At this location, as in Eswatini, open flux coverage may have been periodic, with intermittent heavy doses of harmful UVR, but not subject to prolonged exposure as in western Eurasia. As such, duration of exposure may have been insufficient to warrant investment in tailored clothing, but sufficient to increase demand for ochre sunscreen, an hypothesis that requires further modeling and archaeological testing.

Finally, other intriguing coincidences should be considered as we continue to develop and test hypotheses regarding effects of geomagnetic excursions on humans. Cooccurring with the Laschamps is the earliest known representational cave art—which depicts animals, anthropomorphs, other figures or scenes, as opposed to abstract marks or designs—including images of animals in eastern Borneo, Indonesia (*72*) and western Australia and a hunting scene in southwestern Sulawesi, Indonesia (*73*). Others have noted the occurrence of early rock art during the Laschamps (*26*, and references therein), but we choose to focus specifically on representational art because it presents fewer interpretive hurdles, and lends itself to testable hypotheses regarding biological effects of the excursion on depicted animals and possible human responses. Although more difficult to test empirically, we also note that the earliest known examples of portable art (the Löwenmesche figurine; *74*) and musical instruments (the Geißenklösterle flutes; *75*) also co-occur with the Laschamps. Of course, it is possible that these are simply part of the Aurignacian cultural repertoire, unrelated to the magnetic excursion (i.e., to changes in game availability, visibility of aurora), but testing an hypothesized relationship could prove fruitful. Likewise, two of the earliest known high-altitude sites—Fincha Habera in the Bale Mountains of Ethiopia (~3500 masl; *76*) and Nwya Devu on the Tibetan Plateau (~4600 masl; *77*)—were in use during the Laschamps. Both show evidence of subsistence activities, but their location in world regions where aurora were likely visible during the magnetic excursion suggests the possibility that an additional attraction to these high elevation sites was the view they afforded of, or vertical "proximity" to, aurora.

Global-scale effects of the Laschamps event are likely but more work is necessary to determine their nature, timing, and severity. As a preliminary exploration of empirical evidence that may reflect climatic and biocultural effects of the Laschamps, we have chosen to focus primarily on those areas indicated by our model to have experienced prolonged open flux and auroral coverage, including Western Eurasia where the effects may have lasted three thousand years. The poles were likewise within the region of open flux coverage for much of the event, but evidence for human occupation above 60° N before about 30 ka is currently extremely

limited (*56*). Parts of the Americas experienced auroral and open flux coverage during the Laschamps, but there is currently little support for humans having occupied that part of the world prior to the Last Glacial Maximum (26 - 20 ka). West Africa and the Maghreb were also within the region that likely experienced significant and prolonged irradiation but these areas remain relatively poorly known archaeologically, with a particular scarcity of reference to MIS 3. Much more research is necessary to better understand this phenomenon, both in general and in specific references to potential effects of the Laschamps.

Figures

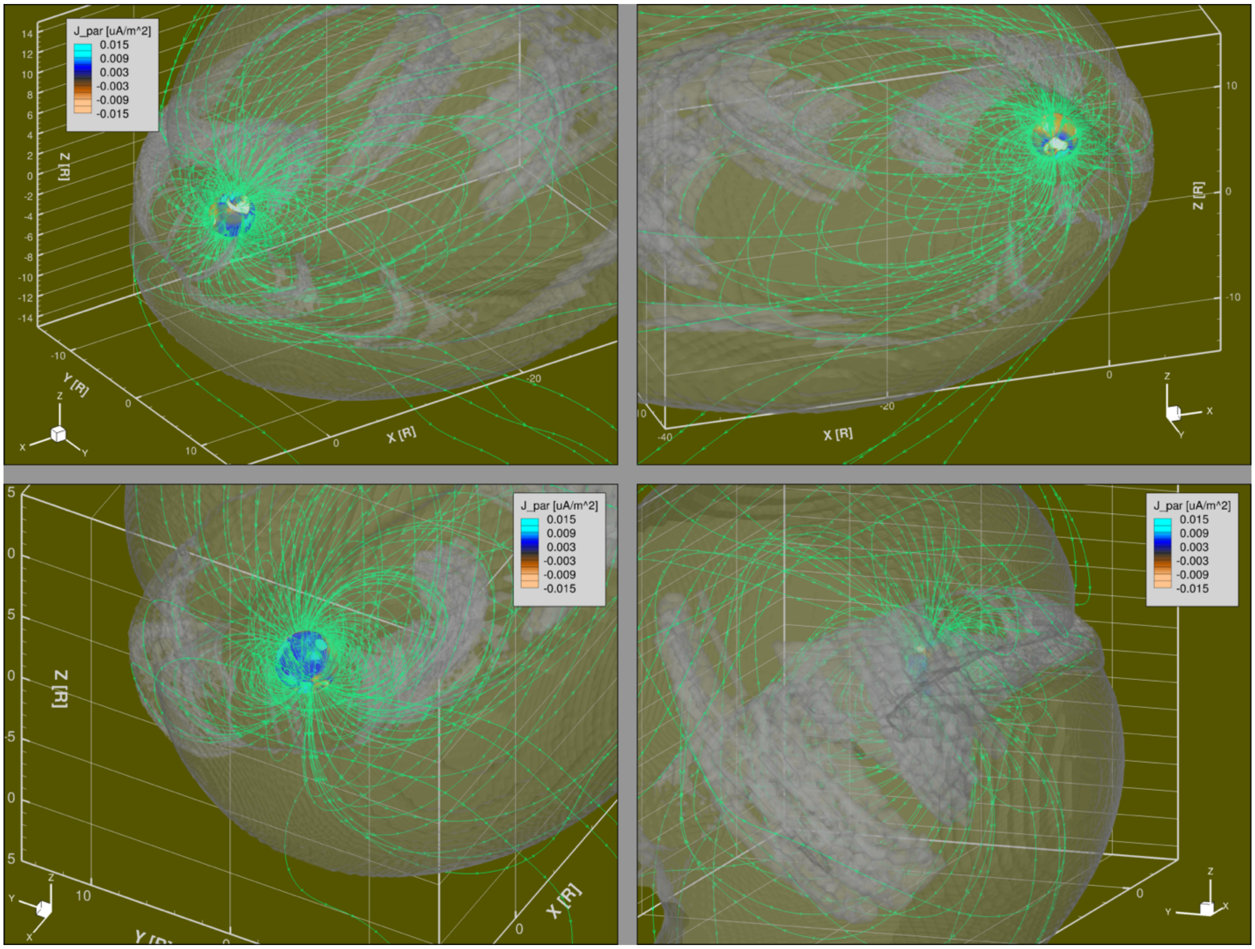

Fig. S1. 3D Magnetospheric configuration at 42.153 ka. During this epoch, the shape and structure of the magnetosphere is predominantly dipolar, while the intrinsic magnetic field is similar to Earth's modern dipolar configuration, albeit quantitatively diminished in value.

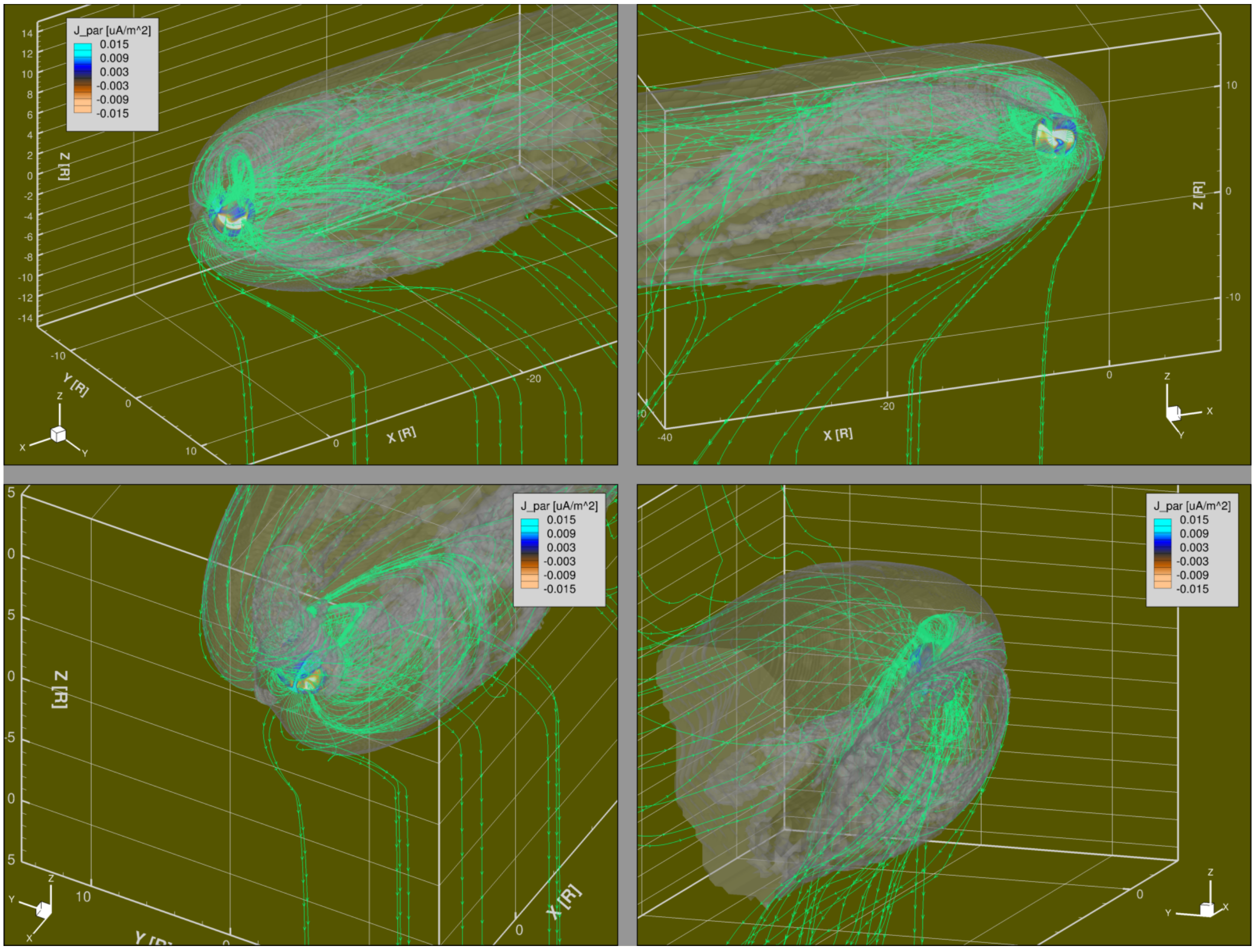

Fig. S2. 3D Magnetospheric configuration at 41.168 ka. During this epoch, the magnetosphere has diminished in size quite significantly while starting to exhibit some tilting in the geomagnetic polar axis, that causes the intrinsic magnetic field near Earth to be more convoluted than the previous epoch.

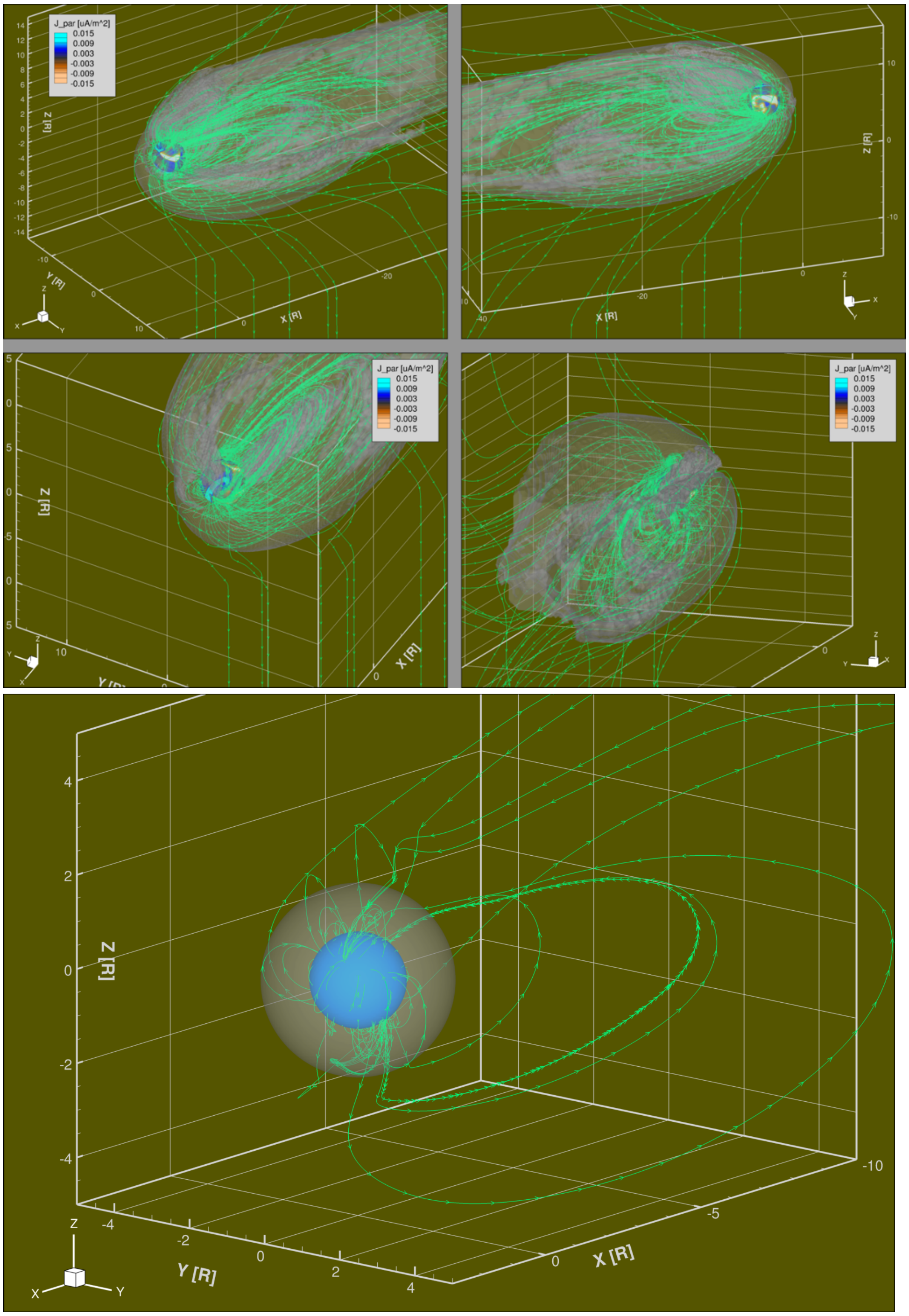

Fig. S3. 3D Magnetospheric configuration at 40.977 ka. During this epoch, the magnetosphere exhibits a diminished magnetosphere, with a day-side magnetosphere that is barely > 2.5 RE. The magnetosphere has a relatively longer night-side, but is almost 2 times smaller than the length of the night-side magnetosphere in the previous epoch.

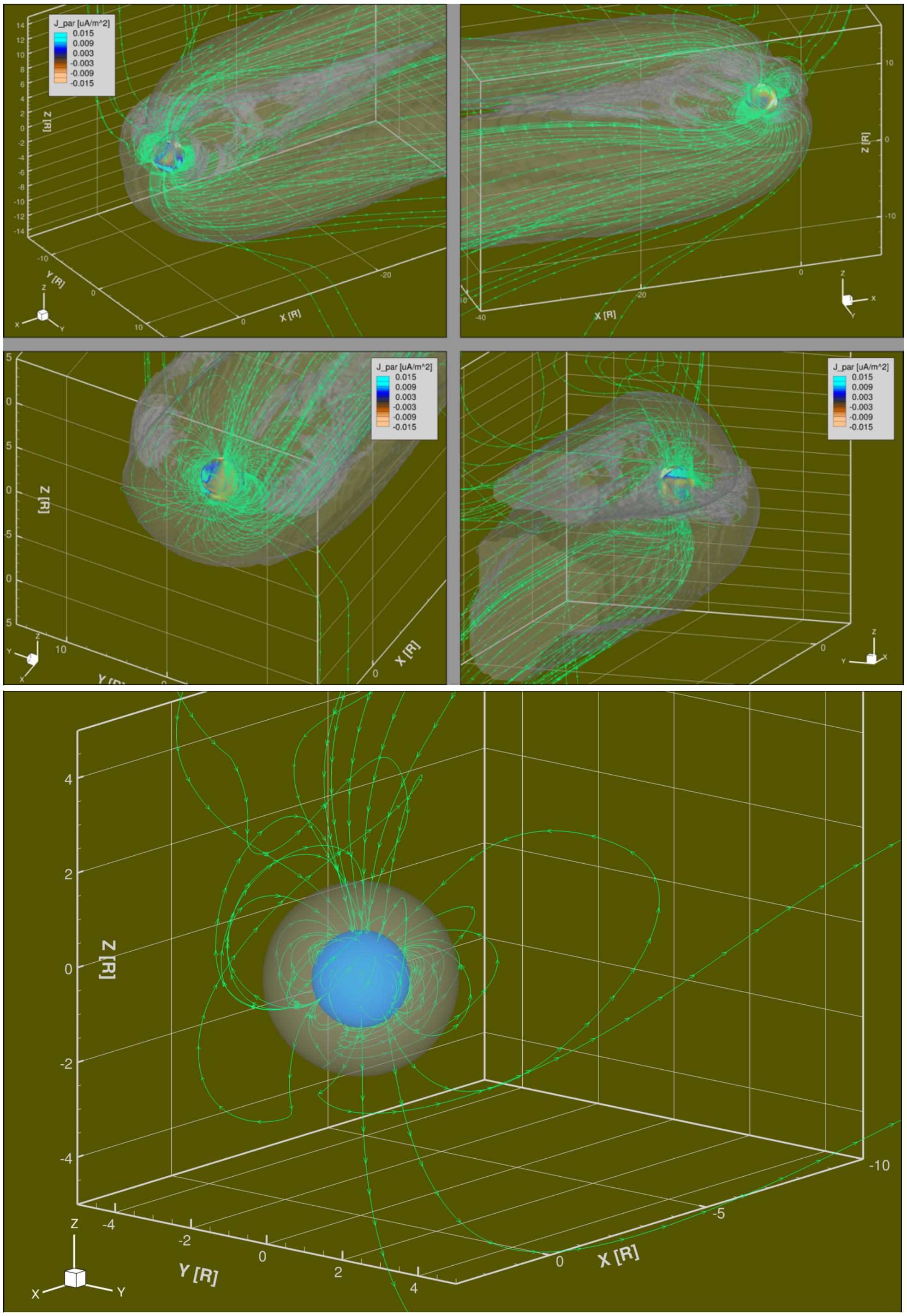

Fig. S4. 3D Magnetospheric configuration at 40.531 ka. The magnetosphere at this epoch slowly retains its dipolar shape, but has huge open field-line regions that close near the Earth. A closer look at the internal structure of the magnetic field shows that the return to a fully dipolar configuration is not complete, and that the magnetic field still retains a significant tilt.

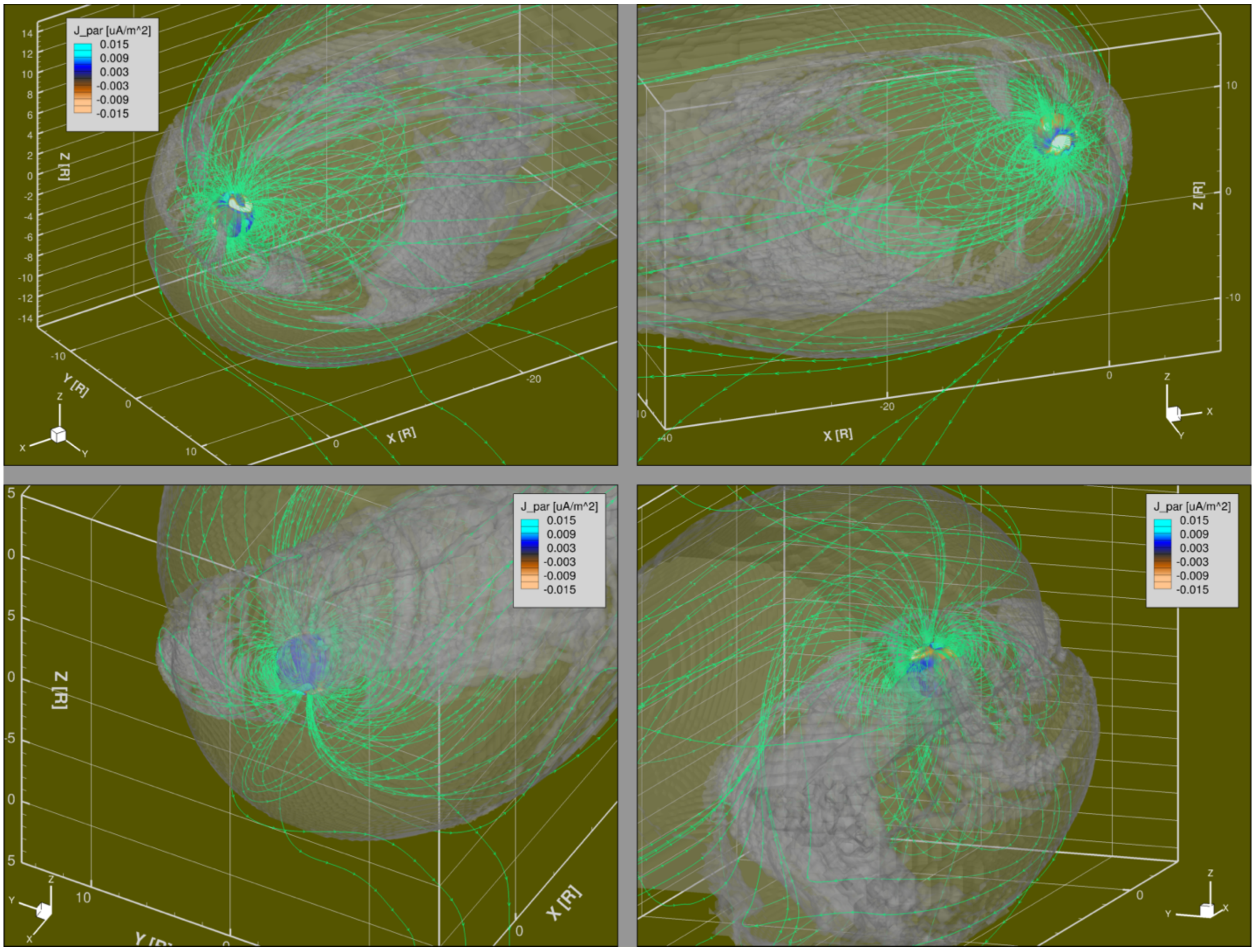

Fig. S5. 3D Magnetospheric configuration at 39.900 ka. During this epoch, the magnetospheric tilt returned to modern-day ranges, with dipole strength slowly recovering. The size and shape of the magnetosphere shows significant similarities to Epoch 1.

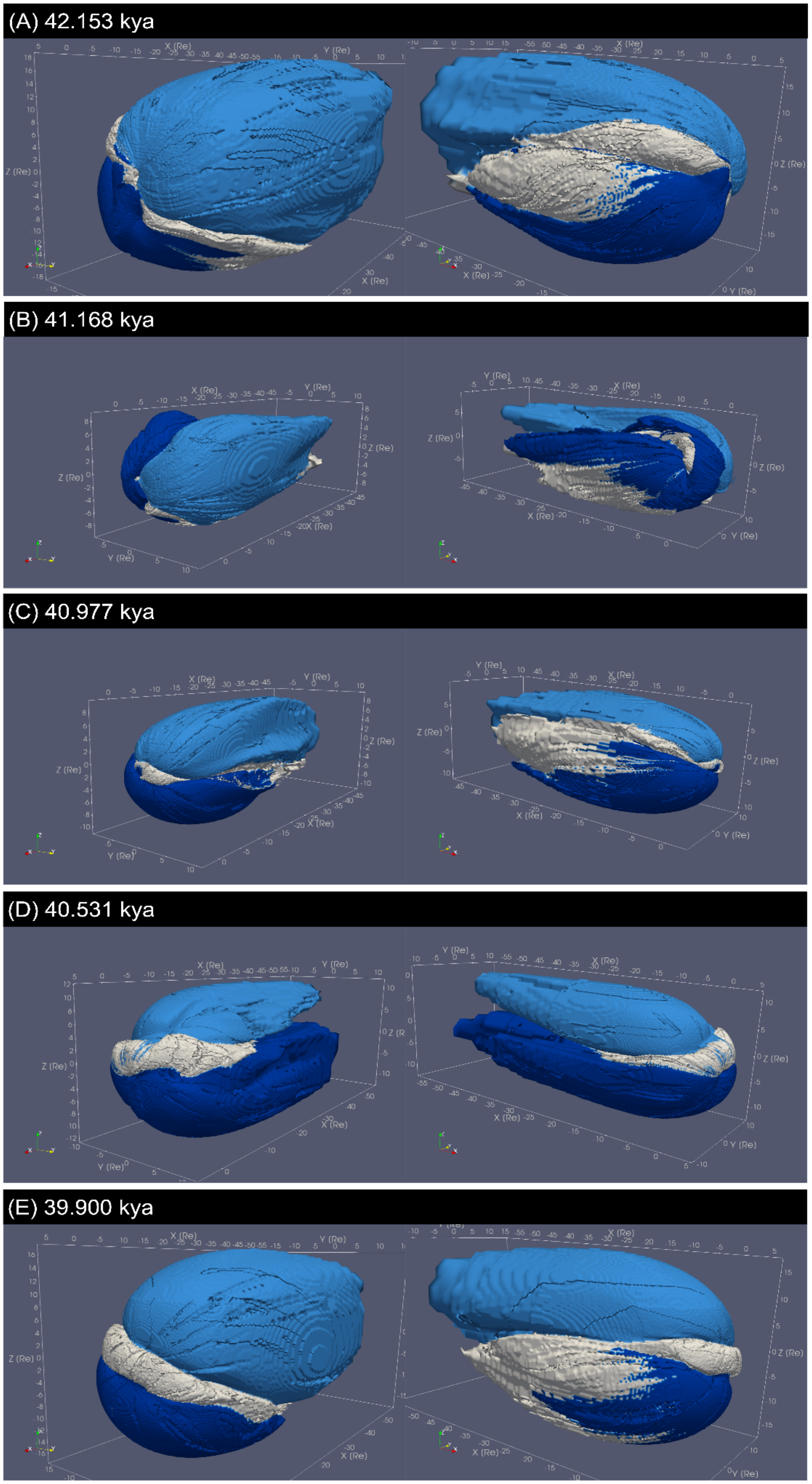
(A) 42.153 kya
(B) 41.168 kya
(C) 40.977 kya
(D) 40.531 kya
(E) 39.900 kya
X (Re)
Y (Re)
Z (Re)

Fig. S6. 3D Magnetopause showing the magnetosphere configuration from 42.153 ka to 39.900 ka. The global configuration of the magnetopause is shown at (A) 42.153 ka, (B) 41.169 ka, (C) 40.977 ka, (D) 40.531 ka, and (E) 39.9 ka. At the beginning and end of this epoch range (42.153, 39.900), the magnetosphere is similar to Earth's modern dipole, with a central closed magnetic field region (in white) between two open magnetic lobes (light and dark blue). In the middle of this epoch range (during the excursion), the magnetosphere becomes significantly diminished and the magnetopause surface topology pattern changes significantly.

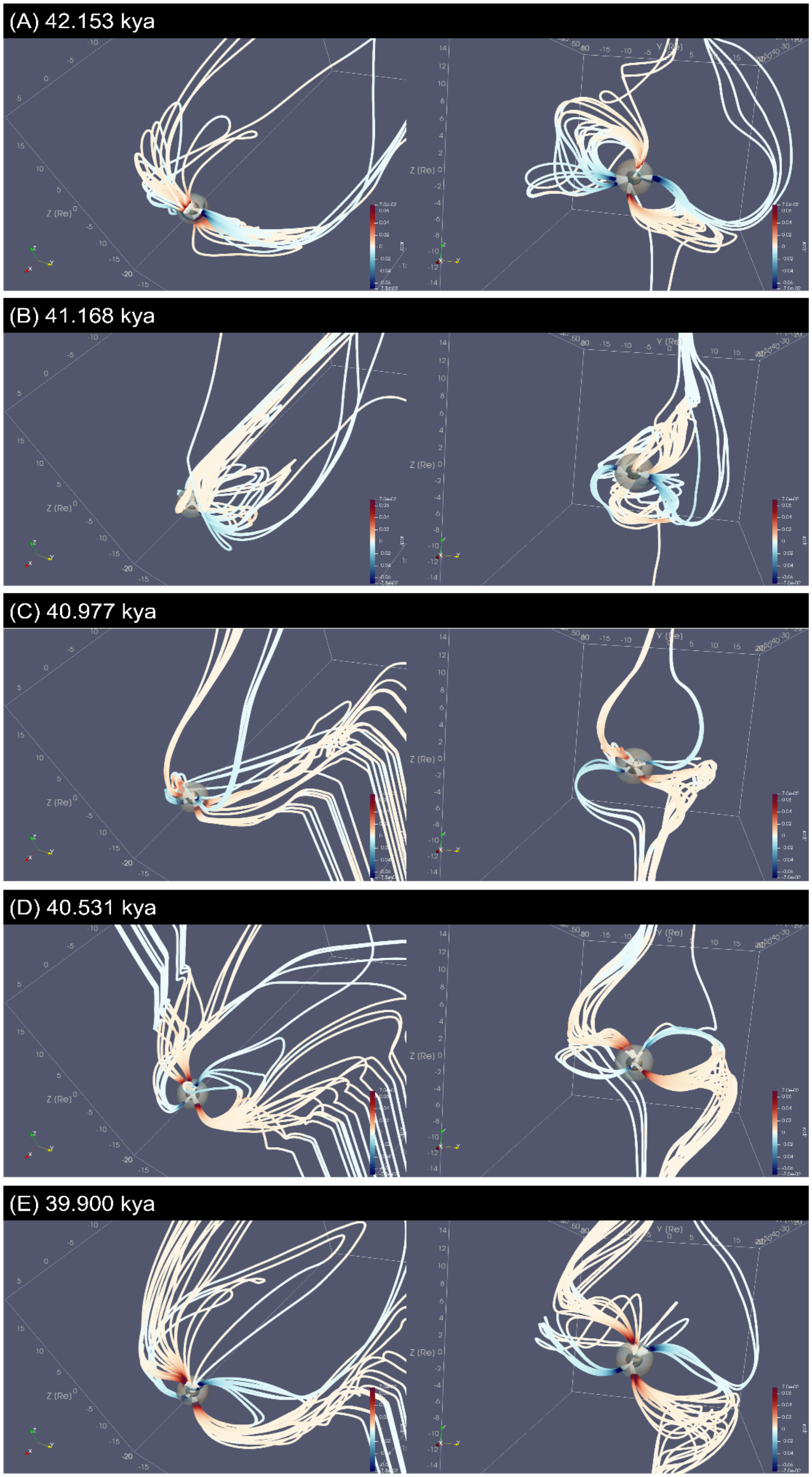
(A) 42.153 kya
(B) 41.168 kya
(C) 40.977 kya
(D) 40.531 kya
(E) 39.900 kya
Z (Re)
Y (Re)

Fig. S7. Magnetic field traced from regions of intense field aligned currents in the magnetosphere configurations from 42.153 ka to 39.900 ka. Traced magnetic field lines are shown at (A) 42.153 ka, (B) 41.169 ka, (C) 40.977 ka, (D) 40.531 ka, and (E) 39.9 ka. The right column panels for the beginning and end of the epoch range show the four part field aligned current structure which is carried by magnetic field extending from Earth out to the solar wind. This pattern would be typical of the magnetosphere with Earth's modern dipole magnetic field. In the middle of this epoch range the magnetic field which carries the strongest field aligned currents changes location and shape.

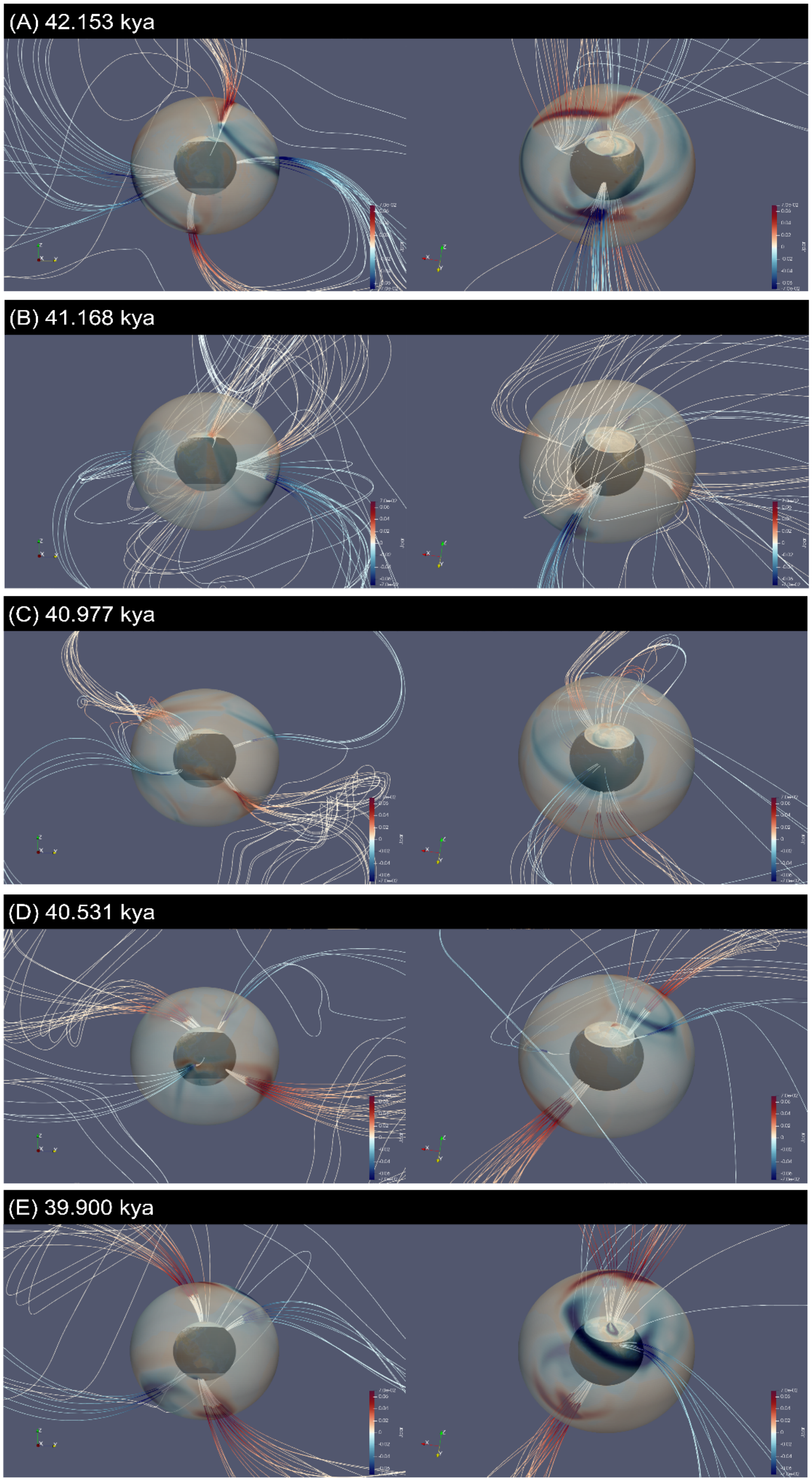
(A) 42.153 kya
(B) 41.168 kya
(C) 40.977 kya
(D) 40.531 kya
(E) 39.900 kya

Fig. S8. Magnetic field traced from regions of intense field aligned currents in the magnetosphere configurations from 39.900 ka to 43.650 ka, with zoomed view. Traced magnetic field lines in the near-Earth region are shown at (A) 42.153 ka, (B) 41.169 ka, (C) 40.977 ka, (D) 40.531 ka, and (E) 39.9 ka. The left column panels for the beginning and end of the epoch range show the four part field aligned current structure which deposits energy to the high latitude regions. This pattern would be typical of the magnetosphere with Earth's modern dipole magnetic field. In the middle of this epoch range the resulting field aligned current connection locations move to lower latitudes and vary in intensity.

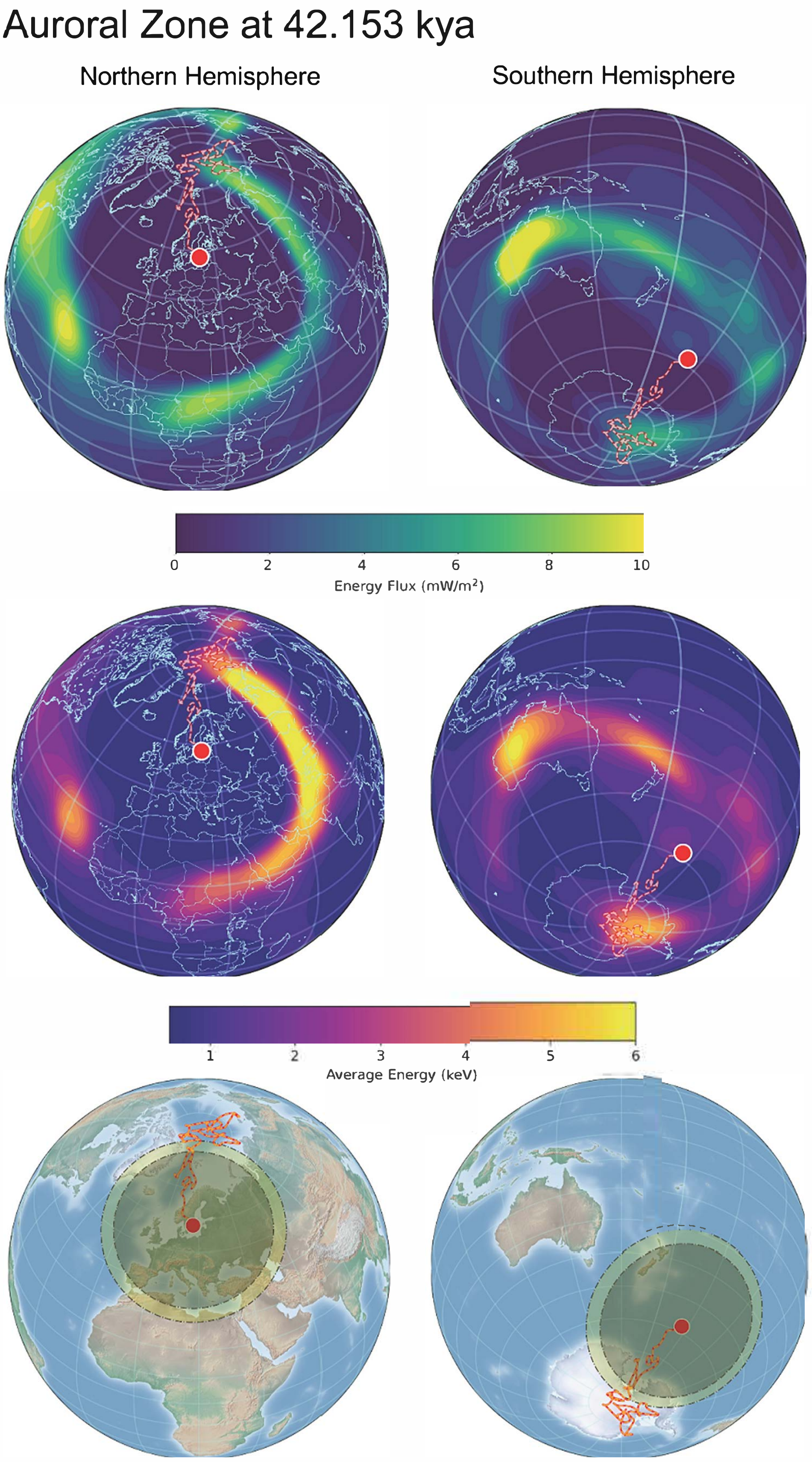
Auroral Zone at 42.153 kya
Northern Hemisphere
Southern Hemisphere
0
2
4
6
8
10
Energy Flux (mW/m²)
1
2
3
4
5
6
Average Energy (keV)

Fig. S9. Auroral Zones in both hemispheres at 42.153 ka. Auroral Energy Flux (Top Row) and Average Energy (Middle Row) at IB, and projected auroral oval and open-closed fieldline boundary extents in Northern and Southern hemispheres (Bottom Row) mapped at 110 kms in geographic coordinates. During this epoch, the auroral zone starts to sharply relocate equatorward, while gradually expanding. Note that these figures do not take into consideration diurnal changes in the magnetic field.

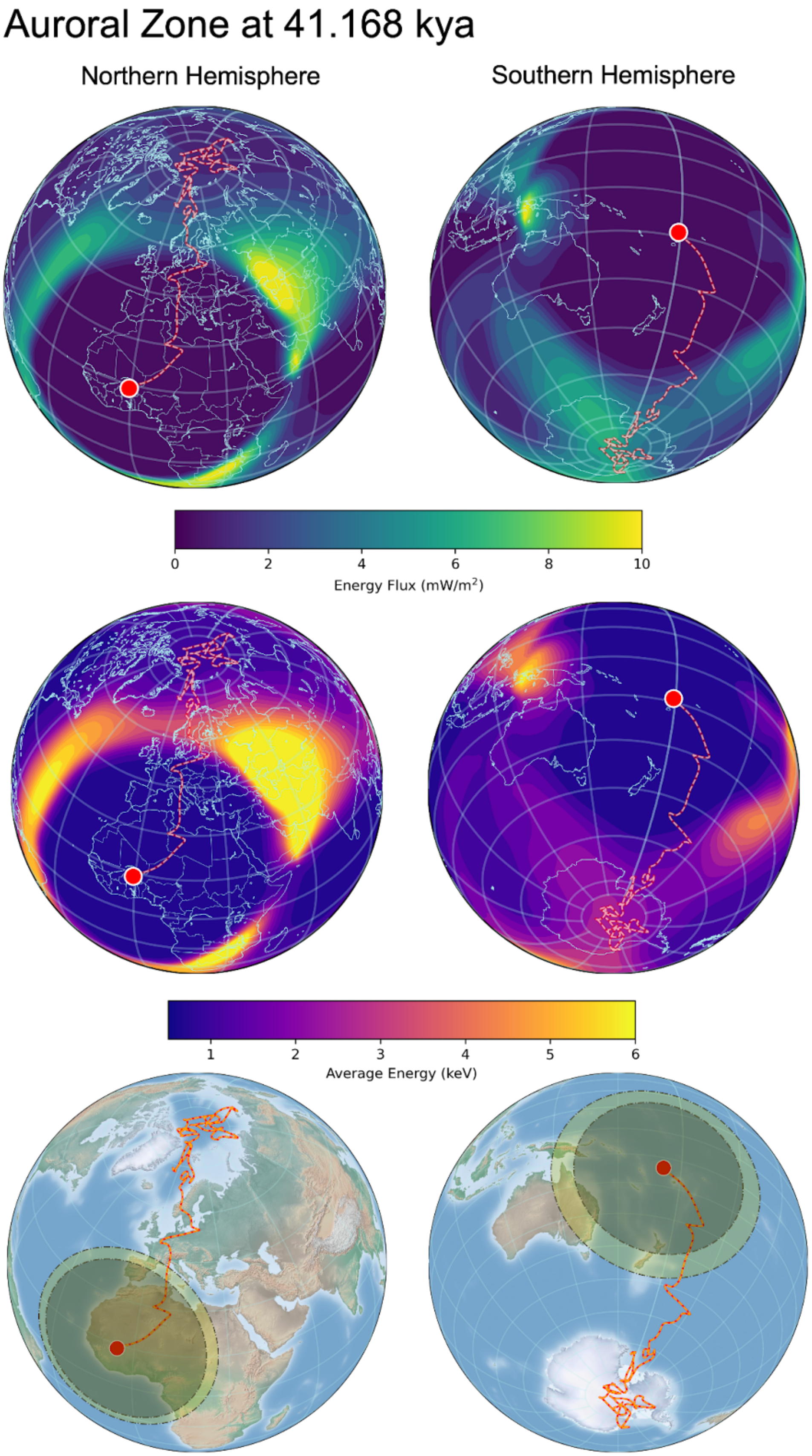

Auroral Zone at 41.168 kya
Northern Hemisphere
Southern Hemisphere
0
2
4
6
8
10
Energy Flux (mW/m²)
1
2
3
4
5
6
Average Energy (keV)

Fig. S10. Auroral Zones in both hemispheres at 41.168 ka. (Top Row) Energy flux, (Middle Row) Average energy, and (Bottom Row) projected auroral and open-closed fieldline boundary extents in Northern and Southern hemispheres. During this epoch, the apparent geomagnetic poles reach their most equatorward positions. In this figure, a significant impact over Europe and Oceania because of auroral activity and open flux regions could be seen.

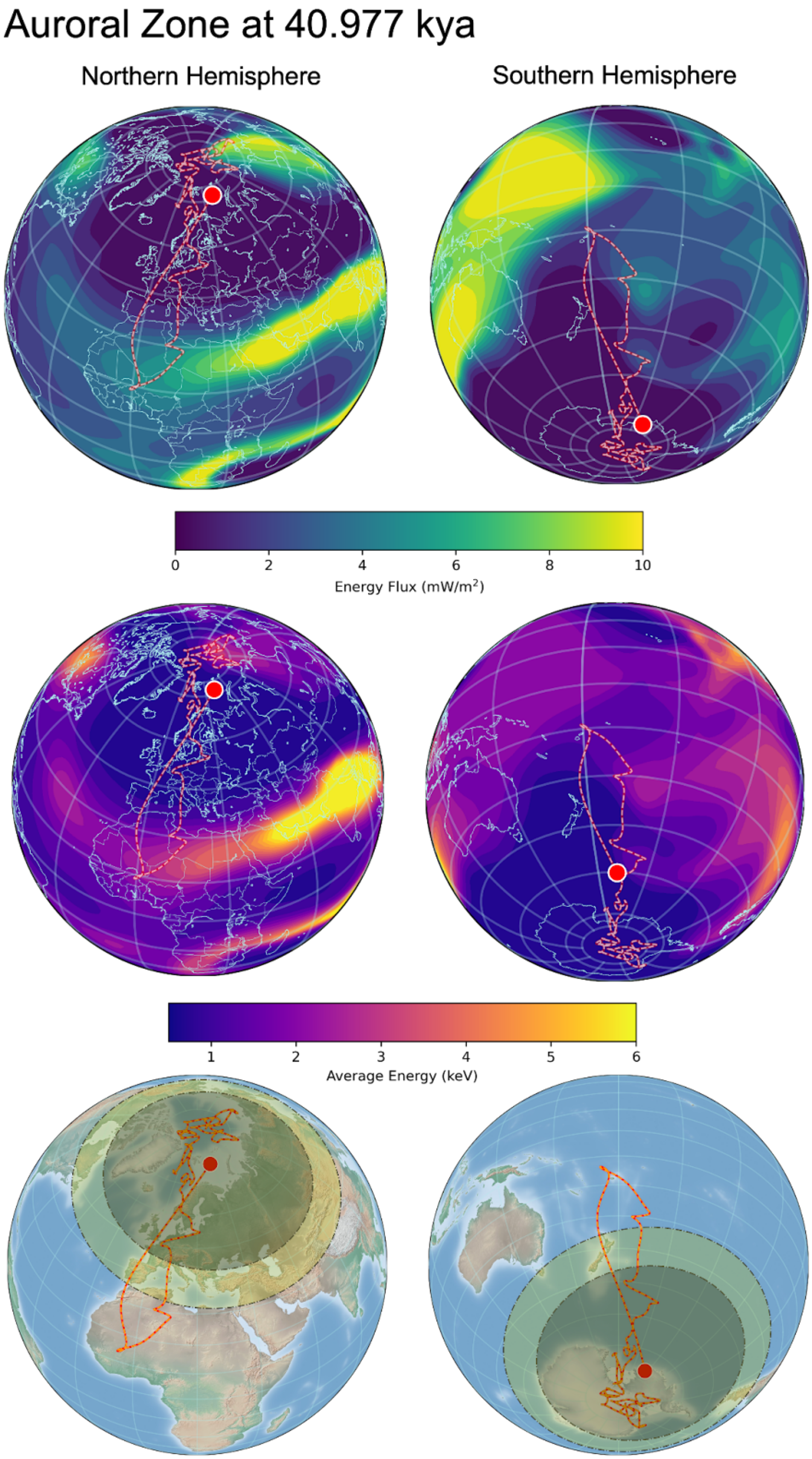

Auroral Zone at 40.977 kya
Northern Hemisphere
Southern Hemisphere
0
2
4
6
8
10
Energy Flux (mW/m^2)
1
2
3
4
5
6
Average Energy (keV)

Fig. S11. Auroral Zones in both hemispheres at 40.997 ka. During this epoch, the apparent geomagnetic poles have relocated poleward. However, auroral expansion across longitudes would most likely have caused global auroral precipitation, along with substantial impact on mid-latitude atmospheric regions by energetic fluxes in the open fieldline region.

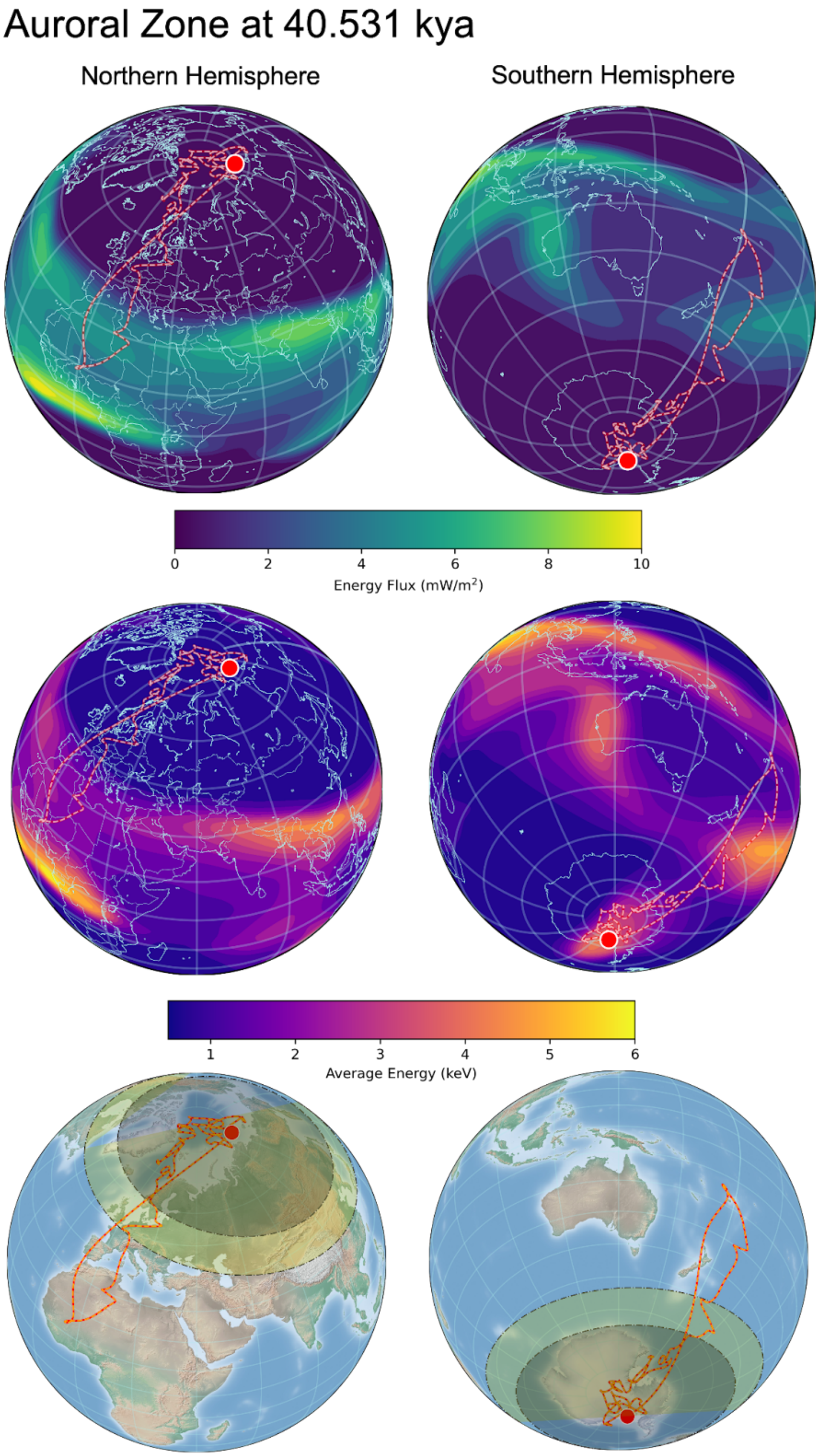
Auroral Zone at 40.531 kya
Northern Hemisphere
Southern Hemisphere
0 2 4 6 8 10
Energy Flux (mW/m^2)
1 2 3 4 5 6
Average Energy (keV)

Fig. S12. Auroral Zones in both hemispheres at 40.531 ka. The state of the auroral region is similar to the previous epoch. However, the geomagnetic condition slowly start to recover as the auroral boundaries shrink in size for the first time since ~43 ka.

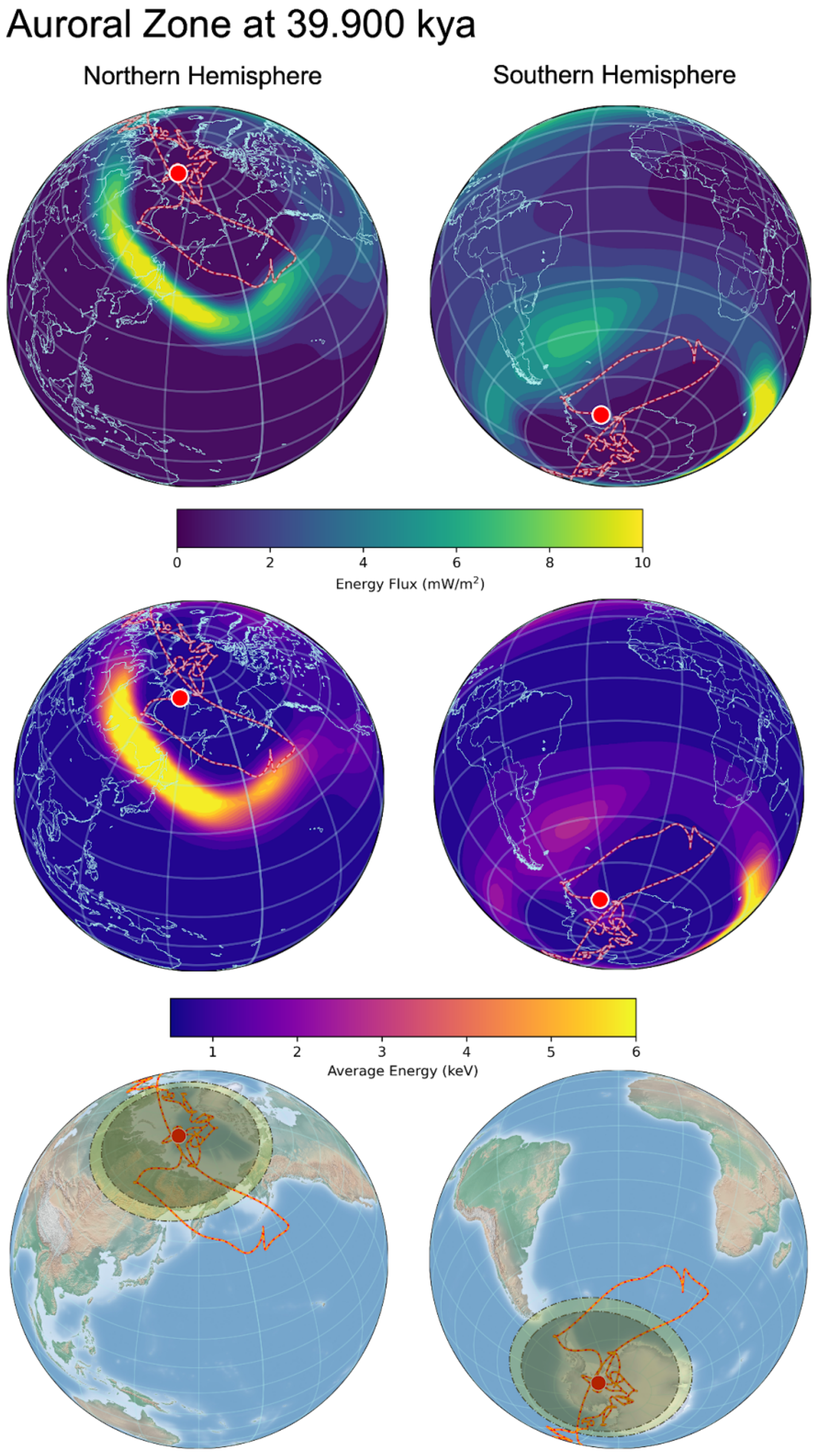
Auroral Zone at 39.900 kya
Northern Hemisphere
Southern Hemisphere
0
2
4
6
8
10
Energy Flux (mW/m²)
1
2
3
4
5
6
Average Energy (keV)

Fig. S13. Auroral Zones in both hemispheres at 39.9 ka. During this epoch, the auroral regions have returned to geographic poles, similar to modern times as the dipole strength slowly recovers over the next few millennia to modern levels.

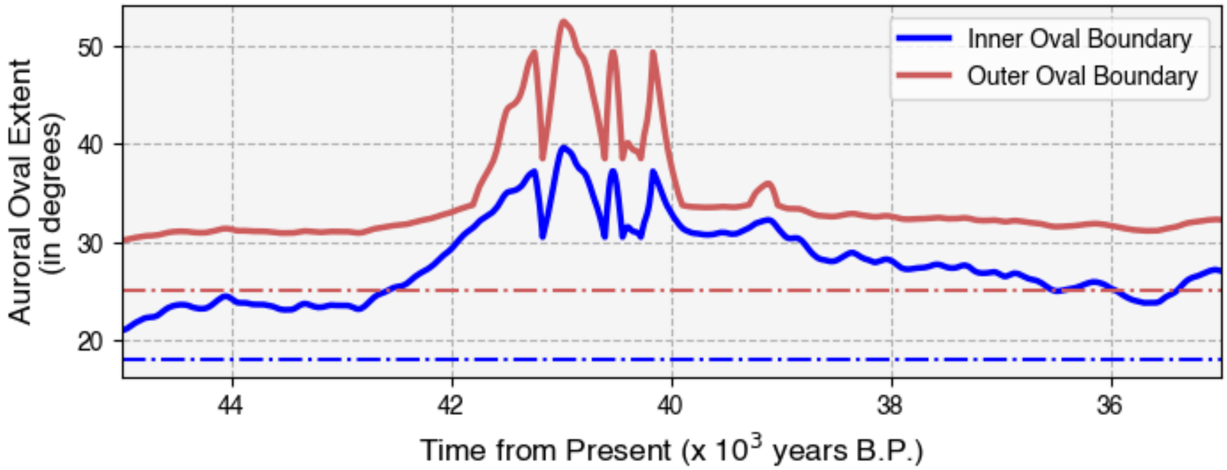

Fig. S14. Auroral Oval Boundaries during the Laschamps Event. Variations in auroral oval boundaries interpolated based on geomagnetic strength and dipole tilt during the Laschamps excursion against the dipole moment variations during the excursion. Note that this interpolation uses a dipole assumption for the auroral zone.

REFERENCES AND NOTES

1. M. Zastrow, "How to improve space weather forecasting," *Eos*, 19 June 2020, p. 131.

2. Y. Kamide, Our life is protected by the Earth's atmosphere and magnetic field: What aurora research tells us. *Biomed. Pharmacother.* **55**, s21–s24 (2000).

3. E. N. Parker, Dynamics of the interplanetary gas and magnetic fields. *Astrophys. J.* **128**, 664 (1958).

4. M. G. Kivelson, C. T. Russell, *Introduction to Space Physics* (Cambridge Univ. Press, 1995).

5. J. W. Dungey, Interactions of solar plasma with the geomagnetic field. *Planet. Space Sci.* **10**, 233–237 (1963).

6. S. Chapman, V. C. A. Ferraro, A new theory of magnetic storms. *Terr. Magn. Atmos. Electr.* **36**, 77–97 (1931).

7. T. I. Gombosi, *Physics of the Space Environment*, Cambridge Atmospheric and Space Science Series (Cambridge Univ. Press, 1998).

8. T. Pulkkinen, Space weather: Terrestrial perspective. *Living Rev. Sol. Phys.* **4**, 1 (2007).

9. R. Schunk, A. Nagy, *Ionospheres: Physics, Plasma Physics, and Chemistry*, Cambridge Atmospheric and Space Science Series (Cambridge Univ. Press, ed. 2, 2009).

10. L. Green, D. Baker, Coronal mass ejections: A driver of severe space weather. *Weather* **70**, 31–35 (2015).

11. J. Liu, W. Wang, L. Qian, W. Lotko, A. G. Burns, K. Pham, G. Lu, S. C. Solomon, L. Liu, W. Wan, B. J. Anderson, A. Coster, F. Wilder, Solar flare effects in the Earth's magnetosphere. *Nat. Phys.* **17**, 807–812 (2021).

12. P. T. Newell, T. Sotirelis, S. Wing, Seasonal variations in diffuse, monoenergetic, and broadband aurora. *J. Geophys. Res. Space Phys.* **115**, A03216 (2010).

13. A. J. Ridley, T. I. Gombosi, D. DeZeeuw, “Ionospheric control of the magnetosphere: Conductance” in *Annales Geophysicae* (Copernicus GmbH, 2004), vol. 22, pp. 567–584.

14. W. Elsasser, E. P. Ney, J. R. Winckler, Cosmic-ray intensity and geomagnetism. *Nature* **178**, 1226–1227 (1956).

15. I. Suter, R. Zech, J. Anet, T. Peter, Impact of geomagnetic excursions on atmospheric chemistry and dynamics. *Clim. Past* **10**, 1183–1194 (2014).

16. J. D. Haigh, The impact of solar variability on climate. *Science* **272**, 981–984 (1996).

17. A. D. Hands, K. A. Ryden, N. P. Meredith, S. A. Glauert, R. B. Horne, Radiation effects on satellites during extreme space weather events. *Space Weather* **16**, 1216–1226 (2018).

18. H. C. Koons, J. F. Fennell, Space weather effects on communications satellites. *URSI Radio Sci. Bull.* **2006**, 27–41 (2006).

19. D. H. Boteler, Space weather effects on power systems. *Geophys. Monogr. Am. Geophys. Union* **125**, 347–352 (2001).

20. G. A. de Wijs, G. Kresse, L. Vočadlo, D. Dobson, D. Alfè, M. J. Gillan, G. D. Price, The viscosity of liquid iron at the physical conditions of the Earth’s core. *Nature* **392**, 805–807 (1998).

21. B. A. Buffett, Onset and orientation of convection in the inner core. *Geophys. J. Int.* **179**, 711–719 (2009).

22. S. Panovska, M. Korte, C. Constable, One hundred thousand years of geomagnetic field evolution. *Rev. Geophys.* **57**, 1289–1337 (2019).

23. D. Gubbins, The distinction between geomagnetic excursions and reversals. *Geophys. J. Int.* **137**, F1–F4 (1999).

24. A. P. Roberts, Geomagnetic excursions: Knowns and unknowns. *Geophys. Res. Lett.* **35**, L17307 (2008).

25. B. S. Singer, A Quaternary geomagnetic instability time scale. *Quat. Geochronol.* **21**, 29–52 (2014).

26. A. Cooper, C. S. M. Turney, J. Palmer, A. Hogg, M. McGlone, J. Wilmshurst, A. M. Lorrey, T. J. Heaton, J. M. Russell, K. McCracken, J. G. Anet, E. Rozanov, M. Friedel, I. Suter, T. Peter, R. Muscheler, F. Adolphi, A. Dosseto, J. T. Faith, P. Fenwick, C. J. Fogwill, K. Hughen, M. Lipson, J. Liu, N. Nowaczyk, E. Rainsley, C. B. Ramsey, P. Sebastianelli, Y. Souilmi, J. Stevenson, Z. Thomas, R. Tobler, R. Zech, A global environmental crisis 42,000 years ago. *Science* **371**, 811–818 (2021).

27. B. S. Singer, B. R. Jicha, N. Mochizuki, R. S. Coe, Synchronizing volcanic, sedimentary, and ice core records of Earth's last magnetic polarity reversal. *Sci. Adv.* **5**, eaaw4621 (2019).

28. J. E. T. Channell, B. S. Singer, B. R. Jicha, Timing of Quaternary geomagnetic reversals and excursions in volcanic and sedimentary archives. *Quat. Sci. Rev.* **228**, 106114 (2020).

29. M. Brown, M. Korte, R. Holme, I. Wardinski, S. Gunnarson, Earth's magnetic field is probably not reversing. *Proc. Natl. Acad. Sci. U.S.A.* **115**, 5111–5116 (2018).

30. K.-H. Glassmeier, J. Vogt, Magnetic polarity transitions and biospheric effects: Historical perspective and current developments. *Space Sci. Rev.* **155**, 387–410 (2010).

31. A. Stadelmann, J. Vogt, K.-H. Glassmeier, M.-B. Kallenrode, G.-H. Voigt, Cosmic ray and solar energetic particle flux in paleomagnetospheres. *Earth Planets Space* **62**, 333–345 (2010).

32. V. S. Airapetian, A. Glocer, G. Gronoff, E. Hébrard, W. Danchi, Prebiotic chemistry and atmospheric warming of early Earth by an active young Sun. *Nat. Geosci.* **9**, 452–455 (2016).

33. J. Vogt, B. Zieger, A. Stadelmann, K.-H. Glassmeier, T. I. Gombosi, K. C. Hansen, A. J. Ridley, MHD simulations of quadrupolar paleomagnetospheres. *J. Geophys. Res. Space Phys.* **109**, A12221 (2004).

34. F. Gong, Y. Yu, K. Bai, J. Cao, Y. Wei, On the particle motion in paleo-magnetosphere during the geomagnetic polarity reversal. *Geophys. Res. Lett.* **50**, e2023GL103843 (2023).

35. S. Panovska, M. Korte, J. Liu, N. Nowaczyk, Global evolution and dynamics of the geomagnetic field in the 15–70 kyr period based on selected paleomagnetic sediment records. *J. Geophys. Res. Solid Earth* **126**, e2021JB022681 (2021).

36. M. Korte, M. C. Brown, S. Panovska, I. Wardinski, Robust characteristics of the Laschamp and Mono Lake geomagnetic excursions: Results from global field models. *Front. Earth Sci.* **7**, 86 (2019).

37. A. Nilsson, N. Suttie, J. S. Stoner, R. Muscheler, Recurrent ancient geomagnetic field anomalies shed light on future evolution of the South Atlantic Anomaly. *Proc. Natl. Acad. Sci. U.S.A.* **119**, e2200749119 (2022).

38. P. Alken, E. Thébault, C. D. Beggan, J. Aubert, J. Baerenzung, W. J. Brown, S. Califf, A. Chulliat, G. A. Cox, C. C. Finlay, A. Fournier, N. Gillet, M. D. Hammer, M. Holschneider, G. Hulot, M. Korte, V. Lesur, P. W. Livermore, F. J. Lowes, S. Macmillan, M. Nair, N. Olsen, G. Ropp, M. Rother, N. R. Schnepf, C. Stolle, H. Toh, F. Vervelidou, P. Vigneron, I. Wardinski, Evaluation of candidate models for the 13th generation International Geomagnetic Reference Field. *Earth Planets Space* **73**, 48 (2021).

39. R. T. Merrill, P. L. McFadden, The geomagnetic axial dipole field assumption. *Phys. Earth Planet. Inter.* **139**, 171–185 (2003).

40. G. Siscoe, C.-K. Chen, The paleomagnetosphere. *J. Geophys. Res.* **80**, 4675–4680 (1975).

41. R. L. McNutt Jr., The magnetospheres of the outer planets. *Rev. Geophys.* **29**, 985–997 (1991).

42. J.-H. Shue, P. Song, The location and shape of the magnetopause. *Planet. Space Sci.* **50**, 549–558 (2002).

43. N. V. Nitta, D. V. Reames, M. L. DeRosa, Y. Liu, S. Yashiro, N. Gopalswamy, Solar sources of impulsive solar energetic particle events and their magnetic field connection to the Earth. *Astrophys. J.* **650**, 438–450 (2006).

44. I. G. Usoskin, M. Korte, G. A. Kovaltsov, Role of centennial geomagnetic changes in local atmospheric ionization. *Geophys. Res. Lett.* **35**, L05811 (2008).

45. Y. I. Feldstein, G. Starkov, The auroral oval and the boundary of closed field lines of geomagnetic field. *Planet. Space Sci.* **18**, 501–508 (1970).

46. L. Mejnertsen, J. P. Eastwood, J. P. Chittenden, A. Masters, Global MHD simulations of Neptune's magnetosphere. *J. Geophys. Res. Space Phys.* **121**, 7497–7513 (2016).

47. J. Y. Lu, Y. Zhou, X. Ma, M. Wang, K. Kabin, H. Z. Yuan, Earth's bow shock: A new three-dimensional asymmetric model with dipole tilt effects. *J. Geophys. Res. Space Phys.* **124**, 5396–5407 (2019).

48. E. A. Lucek, D. Constantinescu, M. L. Goldstein, J. Pickett, J. L. Pinçon, F. Sahraoui, R. A. Treumann, S. N. Walker, The magnetosheath. *Space Sci. Rev.* **118**, 95–152 (2005).

49. P. T. Newell, R. A. Greenwald, J. M. Ruohoniemi, The role of the ionosphere in aurora and space weather. *Rev. Geophys.* **39**, 137–149 (2001).

50. D. F. Smart, M. A. Shea, Fifty years of progress in geomagnetic cutoff rigidity determinations. *Adv. Space Res.* **44**, 1107–1123 (2009).

51. J. Gao, M. Korte, S. Panovska, Z. Rong, Y. Wei, Effects of the Laschamps excursion on geomagnetic cutoff rigidities. *Geochem. Geophys. Geosyst.* **23**, e2021GC010261 (2022).

52. B.-M. Sinnhuber, M. Weber, A. Amankwah, J. P. Burrows, Total ozone during the unusual Antarctic winter of 2002. *Geophys. Res. Lett.* **30**, 1580 (2003).

53. H. Winkler, M. Sinnhuber, J. Notholt, M.-B. Kallenrode, F. Steinhilber, J. Vogt, B. Zieger, K.-H. Glassmeier, A. Stadelmann, Modeling impacts of geomagnetic field variations on middle atmospheric ozone responses to solar proton events on long timescales. *J. Geophys. Res. Atmos.* **113**, D02302 (2008).

54. W. Huang, J. A. Tarduno, T. Zhou, M. Ibañez-Mejia, L. D. Olmo-Barbosa, E. Koester, E. G. Blackman, A. V. Smirnov, G. Ahrendt, R. D. Cottrell, K. P. Kodama, R. K. Bono, D. G.

Sibeck, Y.-X. Li, F. Nimmo, S. Xiao, M. K. Watkeys, Near-collapse of the geomagnetic field may have contributed to atmospheric oxygenation and animal radiation in the Ediacaran Period. *Commun. Earth Environ.* **5**, 207 (2024).

55. R. A. Staff, M. Hardiman, C. B. Ramsey, F. Adolphi, V. J. Hare, A. Koutsodendris, J. Pross, Reconciling the Greenland ice-core and radiocarbon timescales through the Laschamp geomagnetic excursion. *Earth Planet. Sci. Lett.* **520**, 1–9 (2019).

56. V. V. Pitulko, A. N. Tikhonov, E. Y. Pavlova, P. A. Nikolskiy, K. E. Kuper, R. N. Polozov, Early human presence in the Arctic: Evidence from 45,000-year-old mammoth remains. *Science* **351**, 260–263 (2016).

57. C. Stringer, The status of Homo heidelbergensis (Schoetensack 1908). *Evol. Anthropol.* **21**, 101–107 (2012).

58. L. Slimak, C. Zanolli, T. Higham, M. Frouin, J. L. Schwenninger, L. J. Arnold, M. Demuro, K. Douka, N. Mercier, G. Guérin, H. Valladas, P. Yvorra, Y. Giraud, A. Seguin-Orlando, L. Orlando, J. E. Lewis, X. Muth, H. Camus, S. Vandevelde, M. Buckley, C. Mallol, C. Stringer, L. Metz, Modern human incursion into Neanderthal territories 54,000 years ago at Mandrin, France. *Sci. Adv.* **8**, eabj9496 (2022).

59. J. A. Haws, M. M. Benedetti, S. Talamo, N. Bicho, J. Cascalheira, M. G. Ellis, M. M. Carvalho, L. Friedl, T. Pereira, B. K. Zinsious, The early Aurignacian dispersal of modern humans into westernmost Eurasia. *Proc. Natl. Acad. Sci. U.S.A.* **117**, 25414–25422 (2020).

60. International Agency for Research on Cancer (IARC), *Solar and Ultraviolet Radiation*, Monographs on the Evaluation of Carcinogenic Risks to Humans (IARC, 1992), vol. 55.

61. J. J. Bernard, R. L. Gallo, J. Krutmann, Photoimmunology: How ultraviolet radiation affects the immune system. *Nat. Rev. Immunol.* **19**, 688–701 (2019).

62. D. C. Borradale, M. G. Kimlin, Folate degradation due to ultraviolet radiation: Possible implications for human health and nutrition. *Nutr. Rev.* **70**, 414–422 (2012).

63. P. Skoglund, I. Mathieson, Ancient genomics of modern humans: The first decade. *Annu. Rev. Genomics Hum. Genet.* **19**, 381–404 (2018).

64. R. Lozano, M. Naghavi, K. Foreman, S. Lim, K. Shibuya, V. Aboyans, J. Abraham, T. Adair, R. Aggarwal, S. Y. Ahn, M. A. AlMazroa, M. Alvarado, H. R. Anderson, L. M. Anderson, K. G. Andrews, C. Atkinson, L. M. Baddour, S. Barker-Collo, D. H. Bartels, M. L. Bell, E. J. Benjamin, D. Bennett, K. Bhalla, B. Bikbov, A. B. Abdulhak, G. Birbeck, F. Blyth, I. Bolliger, S. Boufous, C. Bucello, M. Burch, P. Burney, J. Carapetis, H. Chen, D. Chou, S. S. Chugh, L. E. Coffeng, S. D. Colan, S. Colquhoun, K. E. Colson, J. Condon, M. D. Connor, L. T. Cooper, M. Corriere, M. Cortinovis, K. Courville de Vaccaro, W. Couser, B. C. Cowie, M. H. Criqui, M. Cross, K. C. Dabhadkar, N. Dahodwala, D. De Leo, L. Degenhardt, A. Delossantos, J. Denenberg, D. C. Des Jarlais, S. D. Dharmaratne, E. R. Dorsey, T. Driscoll, H. Duber, B. Ebel, P. J. Erwin, P. Espindola, M. Ezzati, V. Feigin, A. D. Flaxman, M. H. Forouzanfar, F. G. R. Fowkes, R. Franklin, M. Fransen, M. K. Freeman, S. E. Gabriel, E. Gakidou, F. Gaspari, R. F. Gillum, D. Gonzalez-Medina, Y. A. Halasa, D. Haring, J. E. Harrison, R. Havmoeller, R. J. Hay, B. Hoen, P. J. Hotez, D. Hoy, K. H. Jacobsen, S. L. James, R. Jasrasaria, S. Jayaraman, N. Johns, G. Karthikeyan, N. Kassebaum, A. Keren, J.-P. Khoo, L. M. Knowlton, O. Kobusingye, A. Koranteng, R. Krishnamurthi, M. Lipnick, S. E. Lipshultz, S. L. Ohno, J. Mabweijano, M. F. MacIntyre, L. Mallinger, L. March, G. B. Marks, R. Marks, A. Matsumori, R. Matzopoulos, B. M. Mayosi, J. H. McAnulty, M. M. McDermott, J. McGrath, Z. A. Memish, G. A. Mensah, T. R. Merriman, C. Michaud, M. Miller, T. R. Miller, C. Mock, A. O. Mocumbi, A. A. Mokdad, A. Moran, K. Mulholland, M. N. Nair, L. Naldi, K. M. V. Narayan, K. Nasseri, P. Norman, M. O'Donnell, S. B. Omer, K. Ortblad, R. Osborne, D. Ozgediz, B. Pahari, J. D. Pandian, A. P. Rivero, R. P. Padilla, F. Perez-Ruiz, N. Perico, D. Phillips, K. Pierce, C. A. Pope III, E. Porrini, F. Pourmalek, M. Raju, D. Ranganathan, J. T. Rehm, D. B. Rein, G. Remuzzi, F. P. Rivara, T. Roberts, F. R. De León, L. C. Rosenfeld, L. Rushton, R. L. Sacco, J. A. Salomon, U. Sampson, E. Sanman, D. C. Schwebel, M. Segui-Gomez, D. S. Shepard, D. Singh, J. Singleton, K. Sliwa, E. Smith, A. Steer, J. A. Taylor, B. Thomas, I. M. Tleyjeh, J. A. Towbin, T. Truelsen, E. A. Undurraga, N. Venketasubramanian, L. Vijayakumar, T. Vos, G. R. Wagner, M. Wang, W. Wang, K. Watt, M. A. Weinstock, R. Weintraub, J. D. Wilkinson, A. D. Woolf, S. Wulf, P.-H. Yeh, P. Yip, A. Zabetian, Z.-J. Zheng, A. D. Lopez, C. J. L. Murray, Global and regional mortality from 235

causes of death for 20 age groups in 1990 and 2010: A systematic analysis for the Global Burden of Disease Study 2010. *Lancet* **380**, 2095–2128 (2012).

65. P. Del Fiore, I. Russo, B. Ferrazzi, A. D. Monico, F. Cavallin, A. Filoni, S. Tropea, F. Russano, C. Di Prata, A. Buja, A. Collodetto, R. Spina, S. Carraro, R. Cappallesso, L. Nicole, V. Chiarion-Sileni, J. Pigozzo, L. Dall'Olmo, M. Rastrelli, A. Vecchiato, A. Benna, C. Menin, D. Di Carlo, G. Bisogno, A. P. D. Tos, M. Alaibac, S. Modellin, Melanoma in adolescents and young adults: Evaluation of the characteristics, treatment strategies, and prognostic factors in a monocentric retrospective study. *Front. Oncol.* **11**, 725523 (2021).

66. R. F. Rifkin, L. Dayet, A. Queffelec, B. Summers, M. Lategan, F. d'Errico, Evaluating the photoprotective effects of ochre on human skin by in vivo SPF assessment: Implications for human evolution, adaptation and dispersal. *PLOS ONE* **10**, e0136090 (2015).

67. J. E. T. Channell, L. Vigliotti, The role of geomagnetic field intensity in late quaternary evolution of humans and large mammals. *Rev. Geophys.* **57**, 709–738 (2019).

68. I. Gilligan, *Climate, Clothing and Agriculture in Prehistory* (Cambridge Univ. Press, (2019).

69. M. Collard, L. Tarle, D. Sandgathe, A. Allan, Faunal evidence for a difference in clothing use between Neanderthals and early modern humans in Europe. *J. Anthropol. Archaeol.* **44**, 235–246 (2016).

70. M. Yi, L. Barton, C. Morgan, D. Liu, F. Chen, Y. Zhang, S. Pei, Y. Guan, H. Wang, X. Gao, R. L. Bettinger, Microblade technology and the rise of serial specialists in north-central China. *J. Anthropol. Archaeol.* **32**, 212–223 (2013).

71. R. Garvey, *Patagonian Prehistory: Human Ecology and Cultural Evolution in the Land of Giants* (Univ. of Utah Press, 2021).

72. M. Aubert, R. Lebe, A. A. Oktaviana, M. Tang, B. Burhan, Hamrullah, A. Jusdi, Abdullah, B. Hakim, J. Zhao, I. M. Geria, P. H. Sulistyarto, R. Sardi, A. Brumm, Earliest hunting scene in prehistoric art. *Nature* **576**, 442–445 (2019).

73. M. Aubert, P. Setiawan, A. A. Oktaviana, A. Brumm, P. H. Sulistyarto, E. W. Saptomo, B. Istiawan, T. A. Ma'rifat, V. N. Wahyuono, F. T. Atmoko, J.-X. Zhao, J. Huntley, P. S. C. Taçon, D. L. Howard, H. E. A. Brand, Palaeolithic cave art in Borneo. *Nature* **564**, 254–257 (2018).

74. J. A. Lobell, "New life for the Lion Man," *Archaeology*, 2012, vol. 65, p. 2.

75. T. Higham, L. Basell, R. Jacobi, R. Wood, C. B. Ramsey, N. J. Conard, Testing models for the beginnings of the Aurignacian and the advent of figurative art and music: The radiocarbon chronology of Geißenklösterle. *J. Hum. Evol.* **62**, 664–676 (2012).

76. G. Ossendorf, A. R. Groos, T. Bromm, M. G. Tekelemariam, B. Glaser, J. Lesur, J. Schmidt, N. Akçar, T. Bekele, A. Beldados, S. Demissew, T. H. Kahsay, B. P. Nash, T. Nauss, A. Negash, S. Nemomissa, H. Veit, R. Vogelsang, Z. Woldu, W. Zech, L. Opgenoorth, G. Miehe, Middle Stone Age foragers resided in high elevations of the glaciated Bale Mountains, Ethiopia. *Science* **365**, 583–587 (2019).

77. X. Zhang, B. B. Ha, S. J. Wang, Z. J. Chen, J. Y. Ge, H. Long, W. He, W. Da, X. M. Nian, M. J. Yi, X. Y. Zhou, P. Q. Zhang, Y. S. Jin, O. Bar-Yosef, J. W. Olsen, X. Gao, The earliest human occupation of the high-altitude Tibetan Plateau 40 thousand to 30 thousand years ago. *Science* **362**, 1049–1051 (2018).

78. B. T. Tsurutani, G. S. Lakhina, R. Hajra, Space weather forecasting: What we know now and what are the current and future challenges. *Nonlinear Process. Geophys. Discuss 2019*, https://doi.org/10.5194/npg-2019-38 (2019).

79. V. Airapetian, R. Barnes, O. Cohen, G. A. Collinson, W. C. Danchi, C. F. Dong, A. D. Del Genio, K. France, K. Garcia-Sage, A. Glocer, N. Gopalswamy, J. L. Grenfell, G. Gronoff, M. Güdel, K. Herbst, W. G. Henning, C. H. Jackman, M. Jin, C. P. Johnstone, L. Kaltenegger, C. D. Kay, K. Kobayashi, W. Kuang, G. Li, B. J. Lynch, T. Lüftinger, J. G. Luhmann, H. Maehara, M. G. Mlynczak, Y. Notsu, R. A. Osten, R. M. Ramirez, S. Rugheimer, M. Scheucher, J. E. Schlieder, K. Shibata, C. Sousa-Silva, V. Stamenković, R. J. Strangeway, A. V. Usmanov, P. Vergados, O. P. Verkhoglyadova, A. A. Vidotto, M. Voytek, M. J. Way, G. P.

Zank, Y. Yamashiki, Impact of space weather on climate and habitability of terrestrial-type exoplanets. *Int. J. Astrobiol.* **19**, 136–194 (2020).

80. P. W. Livermore, R. Hollerbach, C. Finlay, An accelerating high-latitude jet in Earth's core. *Nat. Geosci.* **10**, 62–68 (2017).

81. C. Dong, M. Lingam, Y. Ma, O. Cohen, Is Proxima Centauri b habitable? A study of atmospheric loss. *Astrophys. J. Lett.* **837**, L26 (2017).

82. S. Panovska, C. G. Constable, M. C. Brown, Global and regional assessments of paleosecular variation activity over the past 100 ka. *Geochem. Geophys. Geosyst.* **19**, 1559–1580 (2018).

83. C. Laj, C. Kissel, An impending geomagnetic transition? Hints from the past. *Front. Earth Sci.* **3**, 61 (2015).

84. K. G. Powell, P. L. Roe, T. J. Linde, T. I. Gombosi, D. L. DeZeeuw, A solution-adaptive upwind scheme for ideal magnetohydrodynamics. *J. Comput. Phys.* **154**, 284–309 (1999).

85. G. Tóth, I. V. Sokolov, T. I. Gombosi, D. R. Chesney, C. R. Clauer, D. L. De Zeeuw, K. C. Hansen, K. J. Kane, W. B. Manchester, R. C. Oehmke, K. G. Powell, A. J. Ridley, I. I. Roussev, Q. F. Stout, O. Volberg, R. A. Wolf, S. Sazykin, A. Chan, B. Yu, J. Kóta, Space Weather Modeling Framework: A new tool for the space science community. *J. Geophys. Res.* **110**, A12226 (2005).

86. A. Mukhopadhyay, D. T. Welling, M. W. Liemohn, A. J. Ridley, M. Burleigh, C. Wu, S. Zou, H. Connor, E. Vandegriff, P. Dredger, G. Tóth, Global driving of auroral precipitation: 1. Balance of sources. *J. Geophys. Res. Space Phys.* **127**, e2022JA030323 (2022).

87. E. Y. Hathaway, A. Mukhopadhyay, M. W. Liemohn, T. Keebler, B. J. Anderson, S. K. Vines, R. J. Barnes, Extended metric validation of a semi-physical Space Weather Modeling Framework conductance model on field-aligned current estimations. *Front. Astron. Space Sci.* **11**, 1354615 (2024).

88. J. A. Fedder, S. P. Slinker, J. G. Lyon, R. D. Elphinstone, Global numerical simulation of the growth phase and the expansion onset for a substorm observed by Viking. *J. Geophys. Res. Space Phys.* **100**, 19083–19093 (1995).

89. S. Knight, Parallel electric fields. *Planet. Space Sci.* **21**, 741–750 (1973).

90. M. Fridman, J. Lemaire, Relationship between auroral electrons fluxes and field aligned electric potential difference. *J. Geophys. Res. Space Phys.* **85**, 664–670 (1980).

91. J. Beer, M. Vonmoos, R. Muscheler, "Solar variability over the past several millennia" in *Solar Variability and Planetary Climates* (Springer, 2007), pp. 67–79.

92. A. De Santis, E. Qamili, Geosystemics: A systemic view of the Earth's Magnetic field and the possibilities for an imminent geomagnetic transition. *Pure Appl. Geophys.* **172**, 75–89 (2014).

93. F. J. Pavón-Carrasco, A. De Santis, The South Atlantic anomaly: The key for a possible geomagnetic reversal. *Front. Earth Sci.* **4**, 40 (2016).

94. B. Buffett, W. Davis, M. S. Avery, Variability of millennial-scale trends in the geomagnetic axial dipole. *Geophys. Res. Lett.* **46**, 14450–14458 (2019).

95. Y. Pan, J. Li, On the biospheric effects of geomagnetic reversals. *Natl. Sci. Rev.* **10**, nwad070 (2023).

96. S. A. Campuzano, A. De Santis, F. J. Pavón-Carrasco, M. L. Osete, E. Qamili, New perspectives in the study of the Earth's magnetic field and climate connection: The use of transfer entropy. *PLOS ONE* **10**, e0207270 (2018).

97. N. A. Kilifarska, V. G. Bakhmutov, G. V. Melnyk, *The Hidden Link Between Earth's Magnetic Field and Climate* (Elsevier, 2020).

98. K. S. Crider, Y. P. Qi, L. F. Yeung, C. T. Mai, L. H. Zauche, A. Wang, K. Daniels, J. L. Williams, Folic acid and the prevention of birth defects: 30 years of opportunity and controversies. *Annu. Rev. Nutr.* **42**, 423–452 (2022).

99. I. Ivanov, T. Mappes, P. Schaupp, C. Lappe, S. Wahl, Ultraviolet radiation oxidative stress affects eye health. *J. Biophotonics* **11**, e201700377 (2018).

100. B. L. Diffey, Ultraviolet radiation and human health. *Clin. Dermatol.* **16**, 83–89 (1998).

101. UNESCO, Ngwenya Mines (2008); https://whc.unesco.org/en/tentativelists/5421/ [accessed 02 May 2023].

102. F.-G. Wang, S.-X. Yang, J.-Y. Ge, A. Ollé, K.-L. Zhao, J.-P. Yue, D. E. Rosso, K. Douka, Y. Guan, W.-Y. Li, H.-Y. Yang, L.-Q. Liu, F. Xie, Z.-T. Guo, R.-X. Zhu, C.-L. Deng, F. d'Errico, M. Petraglia, Innovative ochre processing and tool use in China 40,000 years ago. *Nature* **603**, 284–289 (2022).
